# Effects of physical activity and dietary supplement on fat free mass and bone mass density during weight loss – a systematic review and meta-analysis.

**DOI:** 10.12688/f1000research.75539.1

**Published:** 2022-01-05

**Authors:** Anja Roth, Martin Sattelmayer, Chloé Schorderet, Simone Gafner, Lara Allet

**Affiliations:** 1 Bern University of Applied Sciences, Bern, Switzerland; 2 HES-SO Valais Wallis, Leukerbad, Switzerland; 3 HES-SO Valais Wallis, Sion, Switzerland; 4 Geneva School of Health Sciences, Genève, Switzerland; 5 Geneva University Hospitals and Faculty of Medicine, Genève, Switzerland

**Keywords:** weight loss, obesity, fat free mass, body composition, exercise training

## Abstract

**Background**: After a diet- or surgery induced weight loss almost 1/3 of lost weight consists of fat free mass (FFM) if carried out without additional therapy. Exercise training and a sufficient supply of protein, calcium and vitamin D is recommended to reduce the loss of FFM.

**Objective**: To investigate the effect of exercise training, protein, calcium, and vitamin D supplementation on the preservation of FFM during non-surgical and surgical weight loss and of the combination of all interventions together in adults with obesity.

**Methods**: A systematic review was performed with a pairwise meta-analysis and an exploratory network meta-analysis according to the PRISMA statement.

**Results**: Thirty studies were included in the quantitative analysis. The pairwise meta-analysis showed for Exercise Training + High Protein vs. High Protein a moderate and statistically significant effect size (SMD 0.45; 95% CI 0.04 to 0.86), for Exercise Training + High Protein vs. Exercise Training a high but statistically not significant effect size (SMD 0.91; 95% CI -0.59 to 2.41) and for Exercise Training alone vs. Control a moderate but statistically not significant effect size (SMD 0.67; 95% CI -0.25 to 1.60). In the exploratory network meta-analysis three interventions showed statistically significant effect sizes compared to Control and all of them included the treatment Exercise Training.

**Conclusions**: Results underline the importance of exercise training and a sufficient protein intake to preserve FFM during weight loss in adults with obesity. The effect of calcium and vitamin D supplementation remains controversial and further research are needed.

## List of Abbreviations

BIA: Bioelectrical impedance analysis

BMD: Bone mineral density

BMI: Body mass index

CI: confidence interval

DXA: Dual Energy X-ray Absorptiometry

FFM: Fat free mass

SD: Standard deviations

SMD: standardized mean difference

## Introduction

In the past three decades, the prevalence of obesity and overweight has risen substantially. Worldwide, the proportion of adults with overweight or obesity increased between 1980 and 2013 from 28.8% to 36.9% in men, and from 29.8% to 38.0% in women. ^1^ Accordingly, the rising prevalence of overweight and obesity has been described as a global pandemic. ^2^ Treatment options for obesity include conservative interventions (diet and/or exercising) and surgical interventions. A 5-10% reduction in baseline weight is frequently recommended by conservative treatment. ^3^ It was reported in the literature that weight loss in this range has not only has a beneficial impact on several obesity-related health conditions and co-morbidities, but also could be cost-effective. ^4^^–^^6^ The non-surgical approach is the initial treatment and consists of multiple components such as improved nutritional aspects, exercise training, cognitive behavioral therapy and a variety of pharmacotherapies. ^7^ Bariatric surgery is considered when conservative approaches fail and is recommended for individuals with a body mass index (BMI) >35 kg/m ^2^ with serious co-morbidities related to obesity. ^8^ A surgical procedure complements but does not replace behavioral, medical and lifestyle treatments. ^7^ Besides the desired weight loss, management and treatment of obesity should have broader objectives than weight loss alone and should include risk reduction and health improvement. ^7^

A repeatedly stated challenge during weight loss is the undesired decrease of fat-free mass (FFM) such as muscle mass and bone mineral density (BMD). ^9^ This undesirable loss of FFM can have serious consequences for patients. Recent studies, for example, reveal that patients undergoing bariatric surgery typically develop an osteoporosis pattern characterized with bone loss and are therefore at higher risk for fractures than obese or non-obese controls. ^10^ FFM is an important component for basal metabolic rate, regulation of body temperature, preservation of skeletal integrity, functional capacity and quality of life. ^11^ Due to this, preserving or minimizing the loss of FFM while losing fat mass is considered optimal and has been referred to as “high-quality weight loss”. ^12^

Literature has shown that after an excessive diet induced weight loss (≥20% of body weight) 27.8% of lost weight consists of FFM, if carried out without additional therapy. ^11^ The same problem occurs with surgically induced weight loss. Subsequent to a gastric bypass surgery without any other intervention, FFM accounts for only 31.3% of the lost weight. ^11^

Current literature shows the importance of resistance training and/or high impact training and a sufficient supply of calcium and vitamin D intake in order to maintain or reduce the FFM and more specifically the loss of BMD. ^13^ Both endurance- and resistance-type exercises seem to help preserve muscle mass during weight loss. ^9^ Resistance-type exercise additionally improves muscle strength. ^9^ Inadequate protein intake results in loss of FFM, a sufficient protein supply is further recommended. ^9^

A recent survey from England revealed that healthcare professionals who take care of bariatric patients often do not follow recommendations on multivitamin, calcium and vitamin D supplementation. ^14^ Furthermore, there is evidence that 67% of the bariatric surgery patients are not physically active enough to maintain their achieved weight loss (compared to 38% in the non-surgical group). ^15^ Considering these findings, it seems evident that the role of exercise training and dietary supplementations such as protein, calcium and vitamin D during weight loss need to be further investigated and their beneficial effects summarized in order to underline their importance.

Even if there is a well-established body of literature on exercise training and dietary supplementation such as protein, calcium or vitamin D during weight loss, no systematic review and meta-analysis has been carried out to evaluate the effects of these interventions on preserving FFM. The purpose of this systematic review and meta-analysis was to summarize the current evidence on maintaining FFM through exercise training and/or dietary supplementation of protein, calcium, and vitamin D during weight loss in adults. We aimed to calculate the effects of each particular intervention, namely exercise, protein supplementation, calcium supplementation and vitamin D supplementation on the preservation of FFM during weight loss. In addition, we investigated whether the combination of all interventions (overall effect of exercise training, protein, calcium, and vitamin D supplementation) has a more beneficial impact on the maintenance of FFM than each intervention individually. This leads to the following research question:

What effect does exercise training have, with or without dietary supplementation (protein or calcium or vitamin D), on the preservation of fat FFM (BMD and muscle mass) in obese adults who experienced weight loss (operative or conservative)?

We hypothesized that a) exercise training with or without dietary supplementation has a beneficial effect on maintaining FFM during weight loss and b) that the combination of exercise therapy and dietary supplementation has a stronger effect on maintaining FFM than each intervention alone. A systematic review with a pairwise meta-analysis and an exploratory network meta-analysis was performed to test our hypothesis.

## Methods

### Design

A systematic review with a meta-analysis and a network meta-analysis was conducted in accordance with the PRISMA Extension Statement for Reporting of Systematic Reviews Incorporating Network Meta-analyses of Health Care Interventions. ^16^ The study protocol was registered on PROSPERO (registration number: CRD42019134651).

### Eligibility criteria

Included were studies assessing adults (≥ 18 years of age) with overweight or obesity (BMI of 25-29.9 kg/m ^2^ or BMI ≥30 kg/m ^2^ ^17^) undergoing a weight loss (diet- or surgery induced) and without secondary diagnosis limiting their exercise activity (e.g. fractures, cancer, neurological diseases). Considered were randomized controlled trials or clinical trials comparing any type of exercise exercise (aerobic and/or resistance type exercise) alone or in combination with dietary supplementation (protein, calcium and/or vitamin D) with a placebo intervention, controlled comparison intervention or standard care. Studies assessing the FFM and/or BMD and/or muscle mass pre- and post-intervention were included. Only studies in English, German and French were included. Studies that used alternative treatment methods for weight loss (such as drugs) were excluded.

### Information sources

A systematic literature search was performed in the following electronic databases:

• Ovid Medline (date of inception [1946] – 27.08.2020) (RRID:SCR_002185)
• Ovid Embase (date of inception [1974] – 27.08.2020) (RRID:SCR_001650)
• Cochrane Central Register of Controlled Trials (CENTRAL) (date of inception [1996] – 27.08.2020) (RRID: SCR_001650)
• ISI Web of Science (date of inception [1900] – 27.08.2020)

### Search strategy

A search strategy was built using the following keywords: (“weight loss” OR overweight OR obesity OR adiposity OR “body weight changes”) AND (“physical training” OR “physical activity” OR exercise OR “exercise therapy”) AND (“dietary supplements” OR nutritional OR supplementation OR protein OR “amino acids” OR calcium OR “vitamin D”) AND (“body composition” OR “fat free mass” OR “lean mass” OR “bone density” OR “muscle mass”). Keywords and medical subject headings were identified with the assistance of a librarian of the Bern University of Applied Sciences (BFH). The Cochrane highly sensitive filter was used to identify randomized controlled trials. No language restrictions were applied. The search strategy was adapted for each database. A detailed search strategy for the database Ovid MEDLINE can be found in the additional files (see Appendix A). ^70^ Additionally, the bibliographies of relevant reviews and of included studies were examined for further potential studies. The search for all databases was conducted on the 10.10.2019 and again on the 27.08.2020.

### Selection process

The title and abstract of publications found in the electronic databases were screened by two independent investigators (AR and CS). In case of disagreement between the two investigators on the selected articles, a discussion occurred, and a consensus was found. The included studies were imported into the reference management software EndNote X9.3.2.3 (Clarivate Analytics, Philadelphia, US) (RRID:SCR_014001) Imported duplicates were removed. An eligibility assessment was performed based on title and abstract. To be included, the studies had to meet all the inclusion criteria. In cases of uncertainty regarding the content of the article based on title and abstract, the full text was accessed and evaluated. The online platform Covidence was used for simplifying the screening process. Full-text versions of studies meeting the inclusion criteria were retrieved for methodological quality assessment and data extraction.

### Data extraction

The information of each study included in this review was extracted and entered in an excel file in duplicate by one investigator. Data were extracted on study characteristics (e.g. author, year, country, study design, inclusion and exclusion criteria, funding, intervention groups, follow-up time, limitations), participants traits (e.g. sample size in each group, mean age, sex, mean weight, BMI and FFM, muscle mass and BMD at baseline) and study results (outcome data, measurement methods, drop-outs). Missing data was obtained for four studies ^18^^–^^21^ by contacting the study authors. If available, change score means and standard deviations (SD) were extracted. Otherwise, final values were used. SD’s were derived from the 95% confidence intervals for two studies. ^22^^,^^23^ The SD’s for seven studies ^19^^,^^21^^,^^24^^–^^28^ were imputed by using the p-value. To have equal scales, outcome data reported in percentages were proportionally converted into kilograms. ^29^^–^^33^

### Statistical analysis

If only one study was available for a treatment comparison (i.e., statistical pooling was not possible), findings were reported as standardized mean differences (SMD) and corresponding 95% confidence intervals (CI). The minimum number of studies needed to perform a meta-analysis was set to 2 studies, if studies were sufficiently similar, as recommended by Valentine *et al.* ^34^ and by Higgins *et al.* ^35^ The analyses were performed using change scores if possible, otherwise final values were used. ^36^ Where enough studies per treatment comparison and outcome were available and the assumption of transitivity was fulfilled, a network meta-analysis was performed using a frequentist model. The assumption of transitivity was assessed for all studies included into the network meta-analysis. ^37^ The studies had to be similar regarding clinical and methodological aspects with exception of the compared interventions.

For all meta-analyses, a random effect model was chosen, because of clinical and methodological diversity among the included studies. Pairwise meta-analyses were performed using the Meta package ^38^ of the statistical analysis software R (R Core Team, Austria) (RRID:SCR_00195). ^38^ The Netmeta package ^39^ was used for the network meta-analysis. SMD’s were calculated and expressed as Hedges’ g. The DerSimonian-Laird estimator was used to analyze the between study variance (τ ^2^). ^40^ In addition, the Hartung-Knapp-Sidik-Jonkman adjustment for random effects models was applied. ^41^ A meta-regression for the variables age at baseline and BMI at baseline was calculated using a mixed-effects model. ^42^

All outcomes of interest were reported as continuous data. The interpretation of the effect sizes was made according to the Cochrane Handbook. ^43^ A small effect size is indicated as 0.2 to 0.49, a moderate effect size as 0.5 to 0.79 and a large effect size as ≥ 0.8. Statistical heterogeneity between studies was assessed using a Chi ^2^-test and the I ^2^-statistics. The interpretation of those calculations was also made according to the Cochrane handbook. ^43^ Results with a p-value <0.05 were considered as statistically significant. If studies assessed different groups, only data of groups meeting our eligibility criteria were analyzed.

### Risk of bias assessment

To assess the quality of the studies the Revised Cochrane risk-of-bias tool for randomized trials (RoB 2.0), ^44^ the updated version of the most used tool for assessing the risk of bias in randomized trials was used. ^44^ Each criterion was evaluated according to the key questions and finally classified as “low risk”, “some concerns” or “high risk”. The risk of bias assessment was performed after the data extraction by two independent reviewers (AR und CS). Disagreements between reviewers were resolved by consensus. A potential publication bias could not be assessed using funnel plots or statistical test’s such as Egger’s test as such methods possess too low power to distinguish chance from real asymmetry when there are less than 10 studies involved in a pairwise meta-analysis. ^43^

## Results

**Figure 1.**
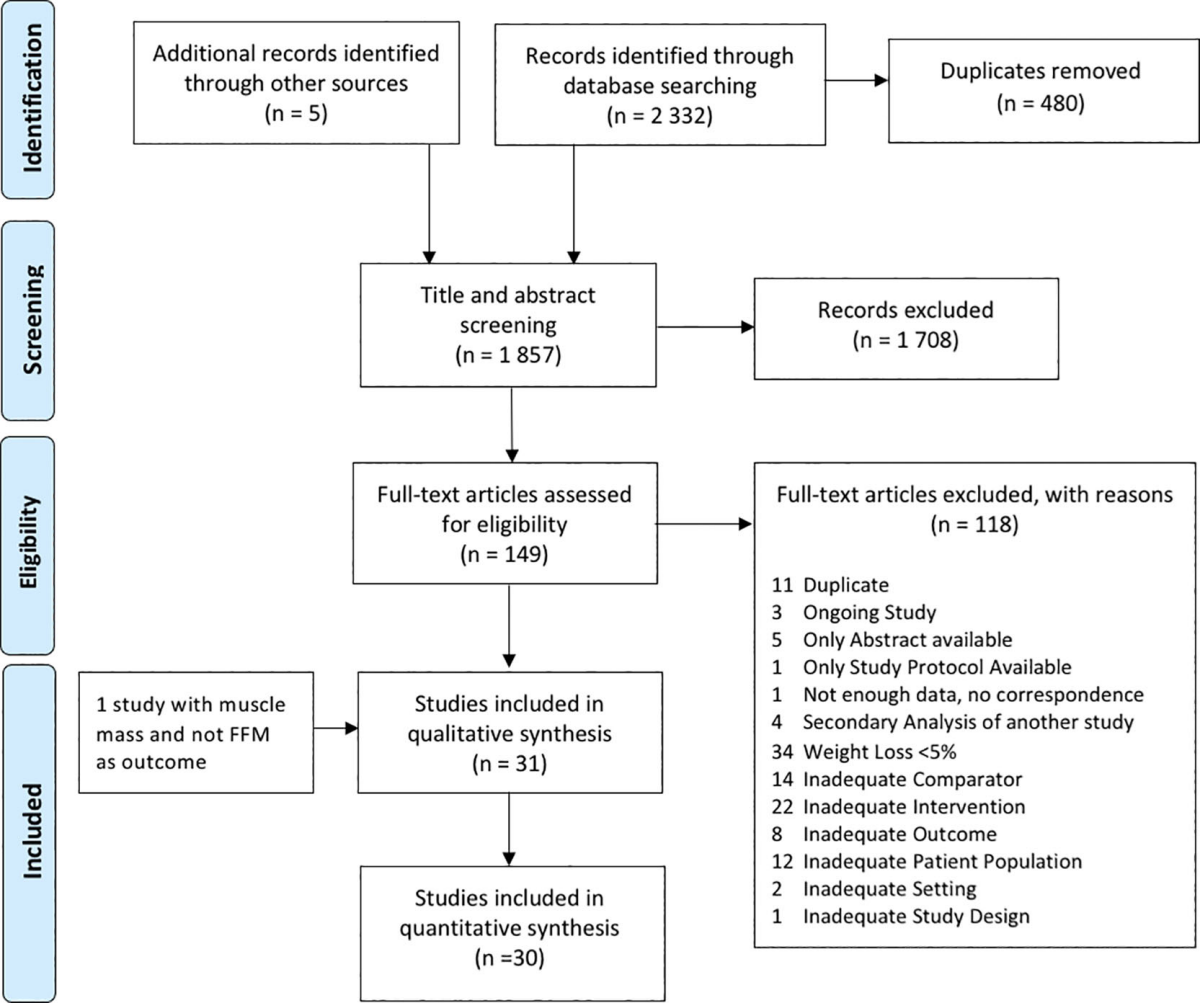
PRISMA flow chart describing the selection process of articles.

Thirty-one studies were eligible, ^69^ but quantitative synthesis was only possible for 30 of those. One study only reported muscle mass and not FFM as the outcome, therefore could not be included in any comparison with another study. ^45^ The study selection process is summarized in Figure 1. A list of all included studies and a table of the characteristics of each study is presented in the additional files (see Appendix B and C). ^71^^,^^72^ The included articles that were published between 1999 and 2019 with sample sizes ranging from 5 to 169 subjects. Ages of the participants ranged from 21 to 74 years and BMIs ranged from 25.8 to 56.8 kg/m ^2^. The length of the follow-up period ranged from 4 weeks to 24 months. Most of the trials were from the USA (k = 11), ^19^^,^^20^^,^^22^^,^^24^^,^^30^^,^^32^^,^^33^^,^^46^^–^^49^ followed by Canada (k = 5) ^25^^,^^50^^–^^53^ and Brazil (k = 3). ^18^^,^^21^^,^^26^ Six studies used resistance training for Exercise Training intervention, ^25^^,^^32^^,^^45^^,^^53^^–^^55^ eight used aerobic training ^12^^,^^23^^,^^33^^,^^46^^,^^49^^,^^56^^–^^58^ and 17 used combined training programs. ^18^^–^^22^^,^^24^^,^^26^^–^^31^^,^^47^^,^^48^^,^^50^^–^^52^

### Risk of bias assessment

**Figure 2.**
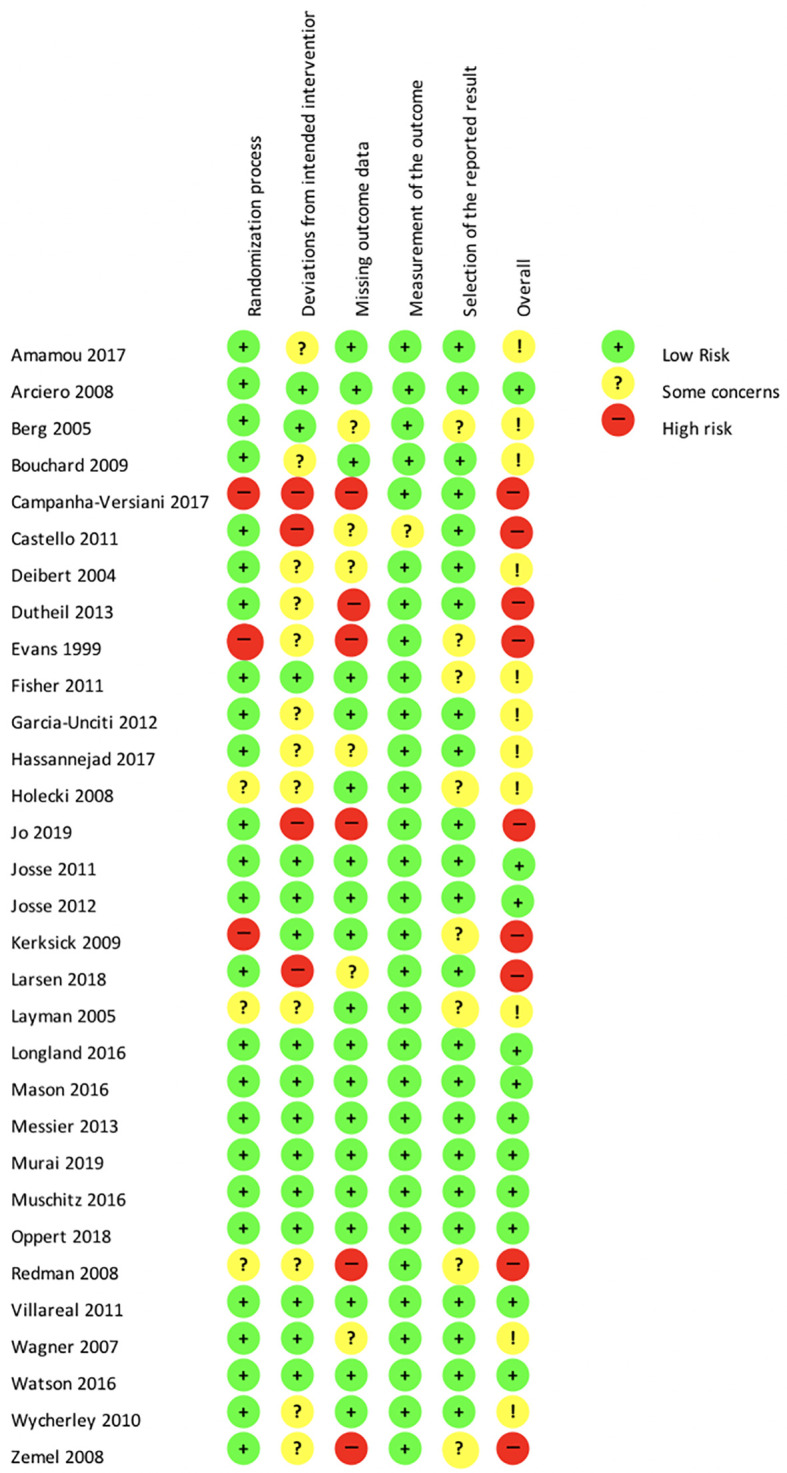
Risk of bias according to revised Cochrane risk-of-bias tool for randomized trials (RoB 2.0).

Figure 2 presents the detailed results of the risk of bias assessment. The randomization process was clearly described in 73.3% of the studies. Deviations from the intended interventions were not clearly described or not appropriately analyzed in 53.3% of the studies. Missing outcome data were reported properly in 56.7% of the studies. The measurement of the outcome data was reliable and valid in 96.7% of all studies. 20% of the included studies are at stake for a potential selective reporting bias. Fifty percent of the included studies in the network meta-analysis were conducted without mentioning sponsors or funding resources.

### FFM

#### Effect of diet induced weight loss on FFM

There were 23 pairwise comparisons with a total of 1642 patients assessing the effect on the outcome FFM. All participants underwent a diet induced weight loss. The commonest comparison was Exercise Training versus Control (k = 7), ^20^^,^^25^^,^^31^^–^^33^^,^^46^^,^^55^ followed by Exercise Training + High Protein versus Exercise Training (k = 6) ^12^^,^^22^^,^^50^^,^^52^^,^^54^^,^^57^ and Exercise Training + High Protein versus High Protein (k = 5). ^24^^,^^30^^,^^53^^,^^56^^,^^58^

**Figure 3.**
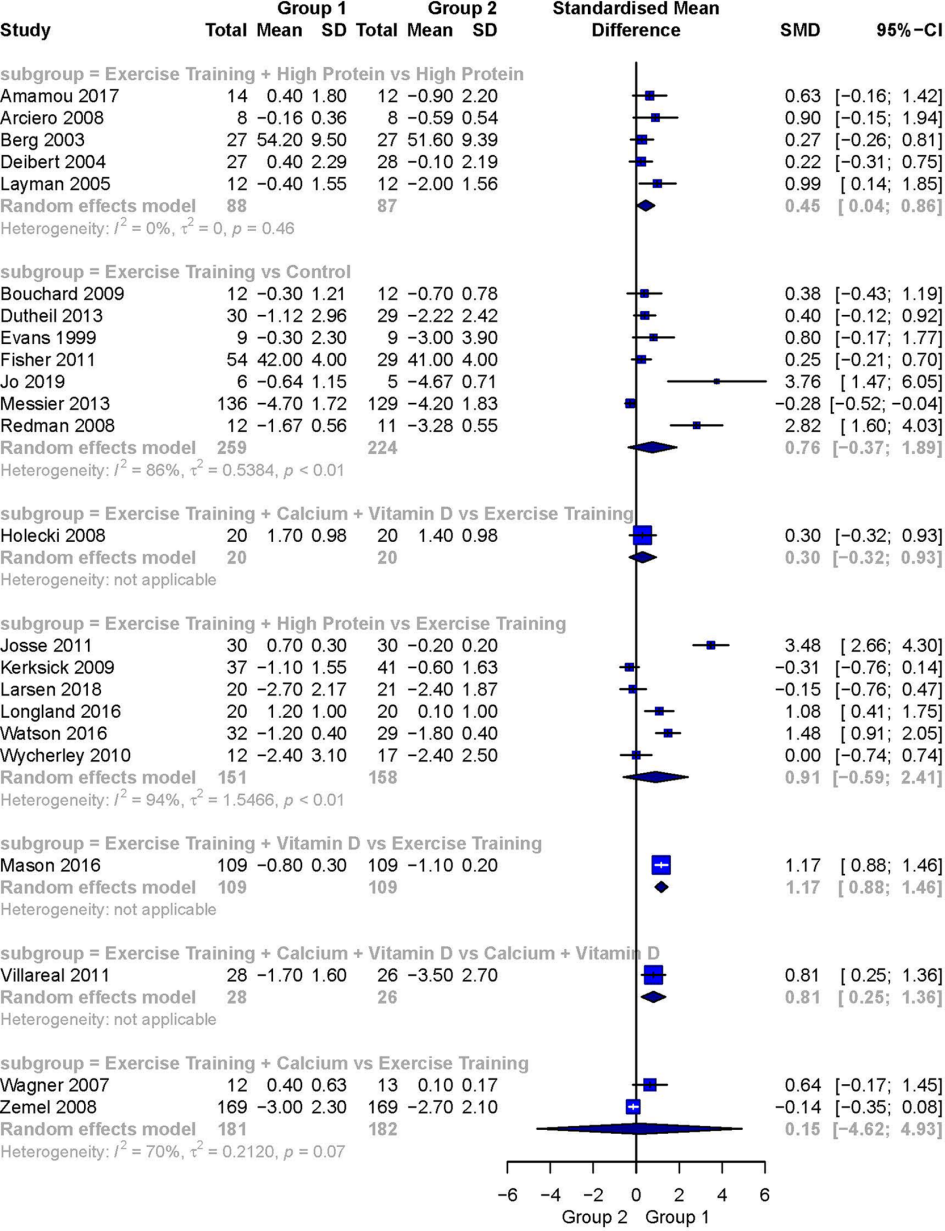
Forest plot for head-to-head comparisons for the outcome fat free mass (kg) during diet induced weight loss. Data are presented as standardized mean differences with 95% CI. The outcome FFM is expressed as change scores and final values.

Figure 3 presents the forest plot of the pairwise meta-analysis. The analysis of the comparison Exercise Training + High Protein versus High Protein ^24^^,^^30^^,^^53^^,^^56^^,^^58^ was the only statistically significant comparison including more than one study. It showed a small to moderate weighted effect size favoring Exercise Training + High Protein (SMD 0.45; 95% CI 0.04 to 0.86). It was also the only meta-analysis demonstrating no heterogeneity (I ^2^ = 0%). The comparison Exercise Training versus Control ^20^^,^^25^^,^^31^^–^^33^^,^^46^^,^^55^ showed a moderate but statistically not significant weighted effect size favoring the intervention group (SMD 0.76; 95% CI **−**0.37 to 1.89). The comparison Exercise Training + High Protein versus Exercise Training ^12^^,^^22^^,^^50^^,^^52^^,^^54^^,^^57^ resulted in a large but again, not statistically significant weighted effect size favoring the intervention group (SMD 0.91; 95% CI **−**0.59 to 2.41). The between study heterogeneity for these two comparisons was considerable and statistically significant (I ^2^ = 84%, and I ^2^ = 94% respectively). The subgroup Exercise Training + Calcium versus Exercise Training ^19^^,^^49^ showed a small effect size with a wide 95% CI favoring Exercise Training + Calcium (SMD 0.15; 95% CI -4.62 to 4.93). The heterogeneity for this comparison was substantial (I ^2^ = 70%). For the comparison Exercise Training + Calcium + Vitamin D versus Exercise Training ^28^ a small and statistically not significant weighted effect size favoring Exercise Training + Calcium + Vitamin D was detected (SMD 0.30, 95% CI −0.32 to 0.93). Heterogeneity was not applicable. Whereas for the comparison Exercise Training + Calcium + Vitamin D versus Calcium & Vitamin D ^59^ a large and statistically significant weighted effect size favoring Exercise Training + Calcium + Vitamin D was detected (SMD 0.81, 95% CI 0.25 to 1.36). Heterogeneity was not applicable.

The comparison Exercise Training + Vitamin D versus Exercise Training ^47^ showed a large and statistically significant effect size (SMD 1.17; 95% CI 0.88 to 1.46) favoring Exercise Training + Vitamin D. Again, heterogeneity was not applicable.

**Figure 4.**
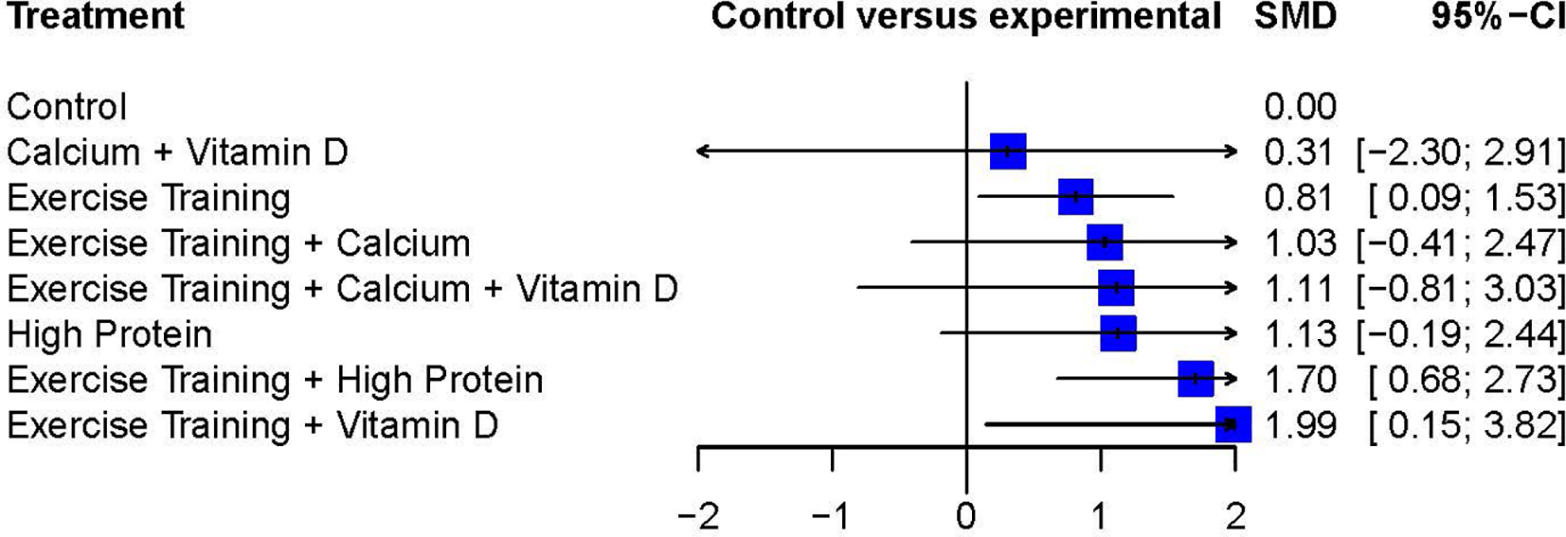
Network meta-analysis ranking and summary of the weighted effect size.

**Figure 5.**
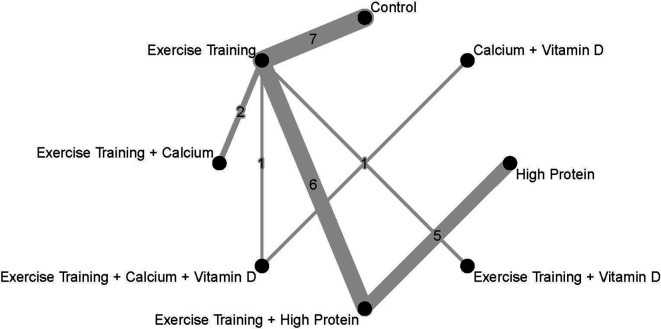
Geometry of the network and number of studies in each comparison. Every intervention is compared to Control. Weighted effect sizes are presented as SMD and corresponding 95%-CI.

In addition to the pairwise meta-analysis, a network meta-analysis was performed for the studies that assessed the outcome FFM after a diet induced weight loss. The treatment Exercise Training + Vitamin D resulted in the highest weighted effect size (SMD 1.99; 95% CI 0.15 to 3.82) and therefore was ranked as the most effective treatment in this network meta-analysis. Followed by Exercise Training + High Protein (SMD 1.70; 95% CI 0.68 to 2.73) and High Protein (SMD 1.13; 95% CI −0.19 to 2.44). Three interventions showed statistically significant weighted effect sizes and all of them included the treatment Exercise Training: Exercise Training + Vitamin D, Exercise Training + High Protein and Exercise Training alone. The treatment Calcium + Vitamin D resulted in a relatively small weighted effect size with a wide confidence interval compared to the other interventions (SMD 0.31, 95% CI −2.30 to 2.91). Figure 4 presents the effect sizes of each treatment compared to the control group and their ranking. The geometry of the network comprised n=8 nodes and n=7 edges. The network did not comprise of any closed loops (i.e. these are parts of the network where all comparisons are connected to each other ^60^). Therefore, it was not possible to explore the inconsistency within the network by comparing direct and indirect treatment estimates as suggested by Veroniki *et al.* ^61^ The network graph with the number of trials is presented in Figure 5. The pooled effect estimations of all direct and network meta-analysis comparisons and the p-scores are presented as well in the additional files (see Appendix D and E). ^73^^,^^74^

A meta-regression was only applicable for the comparison Exercise Training versus Control for the variables age and BMI at baseline. For the variable age at baseline the overall model could explain 15.21% of the variability of the effect sizes and was statistically not significant (R ^2^: 39.41%, p-value: 0.33). There was only a weak relationship between the explanatory variable and the effect estimate (b1: −0.04; 95%CI −0.15 to 0.07, t: −1.11, p: 0.33). For the variable BMI at baseline the overall model could explain 0.00% of the variability of the effect sizes and was statistically not significant (R ^2^: 0.00%, p-value: 0.59). The explanatory variable could not be used as predictor of the effect estimate (b1: 0.1; 95%CI **−**0.37 to 0.57, t: 0.57, p: 0.59).

#### Effect of surgery induced weight loss on FFM

**Figure 6.**
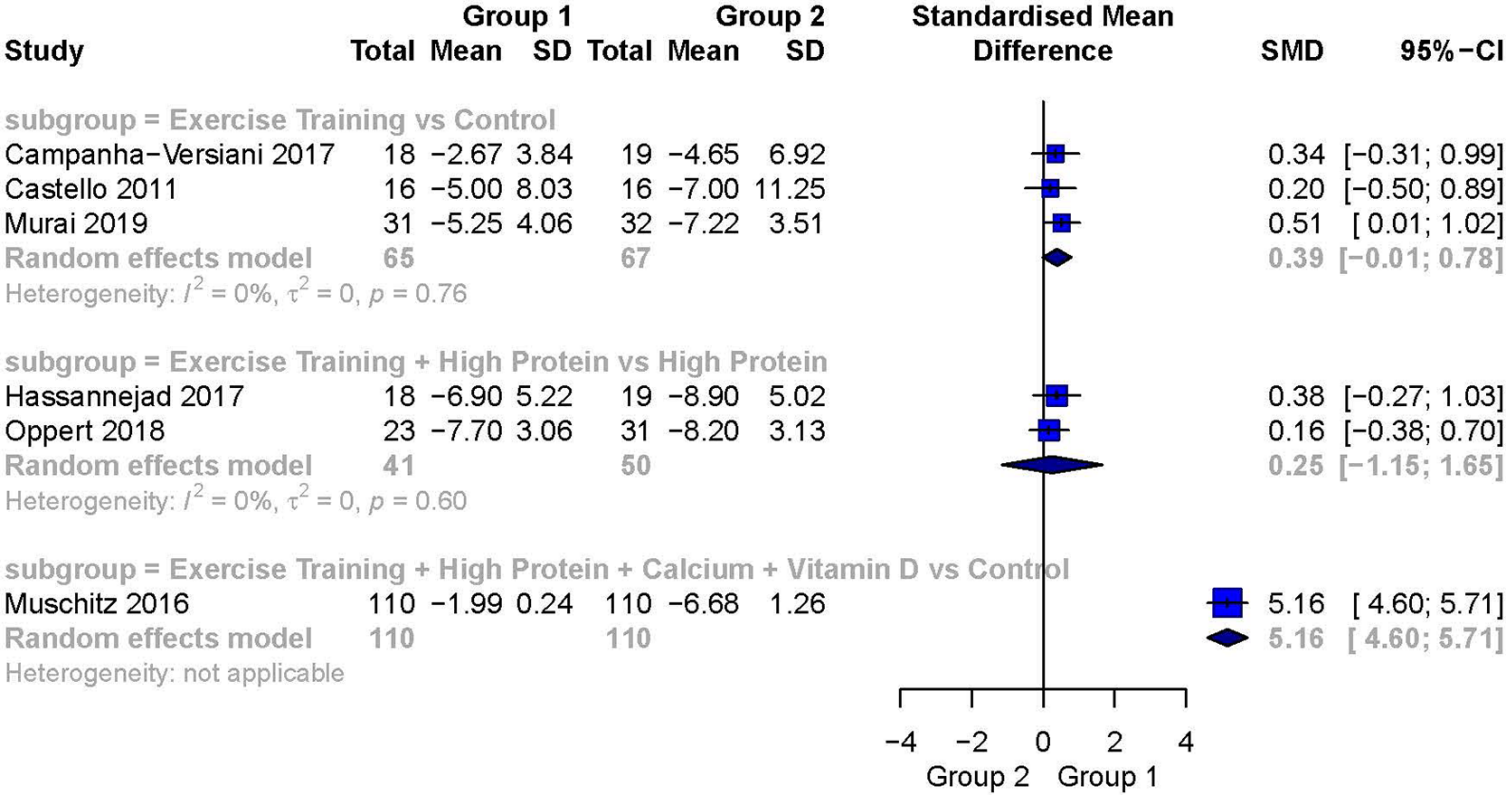
Forest plot for meta-analysis for the outcome change score of fat free mass (kg) during surgery induced weight loss.

Six studies ^18^^,^^21^^,^^23^^,^^26^^,^^27^^,^^29^ that included 443 participants in total reported the change score for FFM during a surgery induced weight loss. Figure 6 represents a summarizing forest plot of the results.

##### Exercise training versus control

Three studies reported FFM as the outcome variable in this subgroup. ^18^^,^^21^^,^^26^ The analysis for this outcome showed a small to moderate weighted effect size in favor of Exercise Training over Control (SMD 0.39; 95% CI −1.01 to 0.78) but the analysis was statistically not significant. There was no evidence of heterogeneity between these studies (I ^2^ = 0%).

##### Exercise training + high protein versus high protein

Two studies reported FFM in this subgroup. ^23^^,^^27^ The analysis for this outcome showed a small weighted effect size favoring Exercise Training + High Protein over High Protein (SMD 0.25; 95% CI −1.15 to 1.65) but the analysis was statistically not significant. There was no evidence of heterogeneity between these studies (I ^2^ = 0%).

##### Exercise training + high protein + calcium + vitamin D versus control

One study reported FFM in this subgroup. ^29^ A very large and statistically significant weighted effect size favoring Exercise Training + High Protein + Calcium + Vitamin D and the control group was detected (SMD 5.16; 95% CI 4.60 to 5.71).

### BMD

#### Effect of diet induced weight loss on BMD

One study investigated BMD during a diet induced weight loss. ^51^ It described a lower amount of BMD-loss at the total-body level in the intervention group. The comparison Exercise Training + High Protein versus Exercise Training ^51^ showed a large weighted effect size (SMD 4.17, 95% CI 3.24 to 5.09) favoring Exercise Training + High Protein.

#### Effect of surgery induced weight loss on FFM

**Figure 7.**
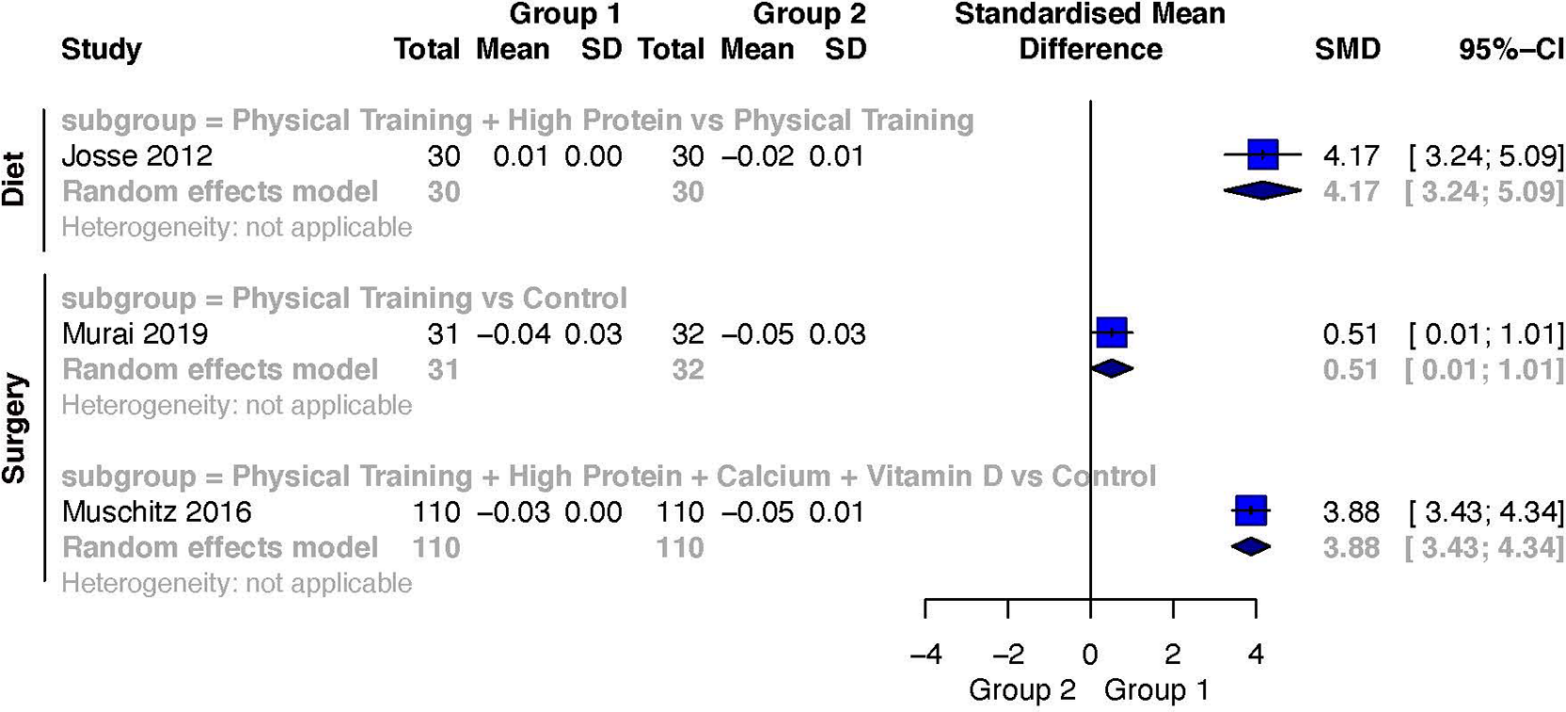
Weighted effects and corresponding 95% CI for the outcome change score in bone mass density at total-body level during diet and surgery induced weight loss.

Two studies investigated BMD after a surgery induced weight loss. ^18^^,^^29^ They described a lower amount of BMD-loss at the total-body level in the intervention group. The comparison Exercise Training versus Control ^18^ resulted in a moderate weighted effect size (SMD 0.51; 95% CI 0.01 to 1.01) favoring Exercise Training. And the comparison Exercise Training + High Protein + Calcium + Vitamin D versus Control ^29^ also resulted in a large, weighted effect size (SMD 3.88; 95% CI 3.43 to 4.34). A forest plot of the results for BMD is presented in Figure 7.

### Muscle mass

One four-arm study including 25 participants assessed muscle mass loss during diet induced weight loss comparing Exercise Training + High Protein versus Control versus Exercise Training versus High Protein. ^45^ No statistically significant difference in muscle mass loss was reported between all groups. Nevertheless, the Exercise Training + High Protein group was the one with the lowest muscle mass loss reported.

## Discussion

The aim of this study was to determine the effect of exercise training, protein, calcium and vitamin D supplementation on the preservation of FFM during weight loss in overweight and obese adults as well as to investigate if the combination of all interventions (overall effect of exercise training, protein, calcium and vitamin D supplementation) has a more beneficial impact on the maintenance of FFM than each single intervention alone. This systematic review with pairwise and network meta-analyses included 2560 participants considered overweight or obese in 31 randomized controlled trials. All the included trials are presented in Appendix B. ^71^ In accordance with our hypothesis, results underline the importance of exercise training and a sufficient protein intake to preserve FFM during weight loss in adults with obesity. The effect of calcium and vitamin D supplementation remains controversial and further research are needed.

Regarding the effect of diet induced weight loss on FFM, results of this study indicate that Exercise Training plus dietary supplementation is superior to only exercise training, to only dietary supplementation and to no additional therapy during weight loss. Results of the pairwise meta-analysis show that the intervention Exercise Training + High Protein was superior in all comparisons and independent of the outcome and type of induced weight loss. Similar findings were reported in earlier research. ^9^

Nevertheless, a heterogeneity could be observed in the results of studies comparing Exercise Training + High Protein versus Exercise Training during diet induced weight loss. This heterogeneity could be partially due to quality differences in the studies. The two studies which favored the exercise training + high protein group to preserve FFM ^52^^,^^57^ were rated as “low risk” for bias, whereas the three studies claiming the contrary were “high risk” for bias or at least showed “some concerns”. ^12^^,^^22^^,^^54^

Results were consistent in favoring exercise training over control during diet induced weight loss although not always statistically significant. These findings are in line with previous reviews. ^9^^,^^11^^,^^15^ Only one included study ^20^ stands out for favoring control over exercise training. In this study the change score for FFM was reported in kg. However, when considering FFM loss in relation to the amount of weight lost, the exercise training group was superior to the control group. ^20^ That being said, reporting only change score of FFM in kg may lead to misinterpretation of study results. Future studies should therefore report both metrics.

Regarding the results of the network meta-analysis, the intervention Exercise Training + Exercise D had the largest weighted effect size on FFM during diet induced weight loss, followed by Exercise Training + High Protein *.* It has to be mentioned that the weighted effect size calculation for Exercise Training + Vitamin D is based on one single study. Researchers and clinicians should therefore be careful with the interpretation of this results.

Regarding the effect of surgery induced weight loss on FFM, studies showed a tendency to favor exercise training over control in the pairwise meta-analysis, but the effect was not statistically significant. Further studies are needed to investigate the effect of exercise training after bariatric surgery on bone and muscle mass and outcomes assessing the exercise function of the participants.

The combination of exercise training, high protein and calcium and vitamin D supplementation seems to be the most effective treatment for the maintenance of FFM during surgery induced weight loss. However, only one relevant study could be found which investigated the combination of all these interventions which limits the informative value.

After our analyses, a new controlled trial investigating the effect of exercise, protein, calcium and vitamin D supplementation during weight loss was published. ^62^ The authors concluded that calcium and vitamin D appeared to have no additional benefit to dietary and exercise interventions in terms of body composition during weight loss. The same researchers discuss though a possible beneficial effect of calcium and vitamin D supplementation for persons who were deficient in these micronutrients prior to supplementation. This might limit the number of people who could benefit from the calcium and vitamin D supplementation. It might further explain why there was such a large effect during surgery induced weight loss in our review, since bariatric surgeries with a malabsorptive component result in a limited nutritional intake. ^9^^,^^11^^,^^13^ As such a surgery induced weight loss is more likely to cause deficiencies in nutrients that are important for FFM (including calcium, vitamin D and protein ^10^) than a dietary induced weight loss. In a meta-analysis by Krieger *et al.* ^63^ a higher daily protein intake of >1.05 to ≤1.20 g/kg body weight was associated with greater FFM maintenance than a lower protein intake of <0.7 g/kg body weight during weight loss. Therefore, the often recommended daily protein intake of 0.8 g/kg body weight may be inadequate for individuals during weight loss. ^63^ Stockton *et al.* reported in their meta-analysis ^64^ that vitamin D supplementation improves muscle function in adults with a vitamin D deficiency but not in non-deficient individuals. Another meta-analysis found a small overall beneficial effect of vitamin D supplementation on BMD at the femoral neck, with larger positive effects in individuals with 25-hydroxyvitamin D levels ≤20 nmol/L. ^65^ Goode *et al.* ^66^ observed a dramatically decreased intestinal calcium uptake and elevated bone resorption markers in patients who underwent gastric bypass even with recommended calcium (1.2 g/d) and vitamin D (8 μg/day) intake. The authors concluded that individuals undergoing bariatric surgery with a malabsorptive component may require even higher dosages to suppress bone loss. However, more research is needed to investigate the question, if individuals following a surgery induced weight loss benefit from calcium and vitamin D supplementation then why don’t non-deficient individuals following a diet induced weight loss?

The methodologies chosen for the estimation of the FFM might influence the FFM values. One review reported that dual energy X-ray absorptiometry (DXA) is the most popular method used, but has also certain biases that may lead to an overestimation of the FFM. ^67^ As almost all included studies (23 out of 29) used DXA to measure FFM we might have an overestimation, but it is unlikely that the method used explains the major differences. Only one study used skinfold measurements for FFM estimation. ^26^ This method relies on the technique and skill of the tester and does not measure FFM *per se,* but rather provides data for calculations to predict FFM based upon body density and fat percentage. ^67^ Three studies assessed FFM using bioelectrical impedance analysis (BIA). ^19^^,^^27^^,^^28^ As this method has inherently large predictive errors, it is insensitive to small improvements in response to treatment. ^67^ Therefore, studies assessing FFM with BIA might have missed small changes in FFM. There is no one-size-fits-all approach for the assessment of FFM in the obese, but future research should be aware of each modality with its benefits and drawbacks and choose the methods appropriate to their situation. ^67^

Major strengths of our study are the large number of included studies (k = 31) and a large sample size (n = 2560). The quality assessment was performed by two independent reviewers which is a further strength. Combining a wide variety of treatments and merging diet- and surgery-induced weight loss strategies leads to an extended overview which adds new knowledge to this research area. However, we are also aware of some limitations of our study. The included studies present heterogeneous samples (e.g., broad age, BMI and follow-up length ranges) and a certain diversity of the exercise training interventions and the supplemental dosages. This might also explain the high heterogeneity in the meta-analyses together with the sometimes very low number of participants in the individual studies. Additionally, the network meta-analysis did not comprise closed loops (i.e., a set of treatments which have been compared against each other). Therefore, it was not possible to analyze the consistency within our network by comparing direct and indirect treatment estimates. ^61^ It should be noted that this network meta-analysis has an exploratory character and therefore should be interpreted with caution. A further statistical limitation represents the fact, that we did not plan a meta-regression from the beginning thus did not report it in the study protocol. It should be interpretated skeptically as there are fewer studies than 10 studies included. ^43^ However, it is of clinical importance that the variables age and BMI at baseline seem to have no influence on the treatment effect. For a more conclusive result, further investigations are needed. The sense of pooling data if only two studies are available can also be questioned but remains in line with current recommendations ^34^^,^^35^. The fact that a network meta-analysis was carried out can also be criticized considering the small number of articles included. However, this method ensures that only comparable data are analyzed together.

Some studies only provided incomplete outcome data, which obligated us to calculate results as prescribed in the methods section. With respect to further empirical trials, research is needed to better identify the effect of the combination of exercise training, protein, calcium, and vitamin D supplementation in obese or overweight patients during dietary or surgically induced weight loss on BMD and muscle mass separately. Additionally, the long-term effect should be addressed and cost effectiveness of exercise interventions and dietary supplementation for obese patients undergoing weight loss examined.

We further have to mention that all types of exercise were classified under “exercise therapy” without distinction between strength training and endurance training even if strength training does not have the same effect than endurance training in the preservation of FFM. ^68^

## Conclusion

The present systematic review with meta-analysis and exploratory network meta-analysis investigated the effect of exercise training, protein, calcium and vitamin D supplementation alone or in all possible combinations on the preservation of FFM during weight loss in adults considered overweight or obese. Results did show a consistent preference of Exercise Training over Control as well as Exercise Training + High Protein over Exercise training alone. These findings underline the importance of exercise training and a sufficient protein intake in order to preserve FFM during weight loss in overweight or obese adults regardless the weight loss approach. The effect of calcium and vitamin D supplementation remains controversial, and it has been hypothesized that only deficient individuals benefit from such intervention. Further research is needed to investigate this hypothesis. The gap in knowledge regarding the combination of all treatments to maintain FFM during weight loss in adults with overweight or obesity could not yet be closed and needs further studies.

## Data availability

### Underlying data

Figshare: DATA SET - EFFECTS OF PHYSICAL ACTIVITY AND DIETARY SUPPLEMENTATION ON FAT FREE MASS AND BONE MASS DENSITY DURING WEIGHT LOSS https://doi.org/10.6084/m9.figshare.17086520. ^69^

The project contains the following underlying data:

- [data_SR_Roth.xlsx] (Raw deidentified data).

*Extended data*

Figshare: Appendix A Search Strategy Medline Ovid https://doi.org/10.6084/m9.figshare.17113475 ^70^

This project contains the following extended data:

- AppendixA_Search_Strategy_Ovid.pdf

Fighare: Appendix B: List of all included studies https://doi.org/10.6084/m9.figshare.17113511 ^71^

This project contains the following extended data:

- AppendixB_List_Included_Studies.pdf

Figshare: Appendix C: Characteristics of studies https://doi.org/10.6084/m9.figshare.17113520 ^72^

This project contains the following extended data:

- AppendixC_Characteristics_of_Studies.pdf

Figshare: Appendix D: Netleague table https://doi.org/10.6084/m9.figshare.17113547 ^73^

This project contains the following extended data:

- AppendixD_netleagueTable.csv

Figshare: Appendix E p-scores:

https://doi.org/10.6084/m9.figshare.17113586 ^74^

This project contains the following extended data:

- AppendixE_PScore.xlsx

Reporting guidelines

The Prisma checklist for this systematic review is available at: https://doi.org/10.6084/m9.figshare.17085932 ^75^

Data are available under the terms of the Creative Commons Zero “No rights reserved” data waiver (CC0 1.0 Public domain dedication).
