# Effects of physical activity and dietary supplement on fat free mass and bone mass density during weight loss – a systematic review and meta-analysis.

**DOI:** 10.12688/f1000research.75539.2

**Published:** 2022-08-12

**Authors:** Anja Roth, Martin Sattelmayer, Chloé Schorderet, Simone Gafner, Lara Allet

**Affiliations:** 1 Bern University of Applied Sciences, Bern, Switzerland; 2 School of Health Sciences, University of Applied Sciences and Arts Western Switzerland, Valais, HES-SO Valais Wallis, Leukerbad, Switzerland; 3 School of Health Sciences, University of Applied Sciences and Arts Western Switzerland, Valais, HES-SO Valais Wallis, Sion, Switzerland; 4 Geneva School of Health Sciences, Genève, Switzerland; 5 Geneva School of Health Sciences, HES‑SO, University of Applied Sciences and Arts Western Switzerland, 47 Avenue de Champel, 1206 Geneva, Switzerland; 6 Geneva University Hospitals and Faculty of Medicine, Genève, Switzerland

**Keywords:** weight loss, obesity, fat free mass, body composition, exercise training

## Abstract

**Background**: After a diet- or surgery induced weight loss almost 1/3 of lost weight consists of fat free mass (FFM) if carried out without additional therapy. Exercise training and a sufficient supply of protein, calcium and vitamin D is recommended to reduce the loss of FFM.

**Objective**: To investigate the effect of exercise training, protein, calcium, and vitamin D supplementation on the preservation of FFM during non-surgical and surgical weight loss and of the combination of all interventions together in adults with obesity.

**Methods**: A systematic review was performed with a pairwise meta-analysis and an exploratory network meta-analysis according to the PRISMA statement.

**Results**: Thirty studies were included in the quantitative analysis. The pairwise meta-analysis showed for Exercise Training + High Protein vs. High Protein a moderate and statistically significant effect size (SMD 0.45; 95% CI 0.04 to 0.86), for Exercise Training + High Protein vs. Exercise Training a high but statistically not significant effect size (SMD 0.91; 95% CI -0.59 to 2.41) and for Exercise Training alone vs. Control a moderate but statistically not significant effect size (SMD 0.67; 95% CI -0.25 to 1.60). In the exploratory network meta-analysis three interventions showed statistically significant effect sizes compared to Control and all of them included the treatment Exercise Training.

**Conclusions**: Results underline the importance of exercise training and a sufficient protein intake to preserve FFM during weight loss in adults with obesity. The effect of calcium and vitamin D supplementation remains controversial and further research are needed.

## List of abbreviations

BIA: Bioelectrical impedance analysis

BMD: Bone mineral density

BMI: Body mass index

CI: Confidence interval

DXA: Dual energy X-ray absorptiometry

FFM: Fat-free mass

SD: Standard deviation

SMD: Standardized mean difference

## Introduction

The global prevalence of obesity and excess bodyweight has risen substantially in the past three decades. Worldwide, between 1980 and 2013, the proportion of overweight or obese adults increased from 28.8% to 36.9% in men and from 29.8% to 38.0% in women. ^1^ The rising prevalence of overweight and obese individuals has been described as a global pandemic. ^2^ Treatment options for obesity include conservative interventions (diet and/or exercise) and surgical interventions. A 5% to 10% reduction in baseline weight is frequently recommended as a conservative treatment. ^3^ The literature reports that weight loss within this range not only has a beneficial impact on several obesity-related health conditions and co-morbidities, but can also be cost-effective. ^4^ ^–^ ^6^ A non-surgical, multi-component approach is generally the initial treatment, including aspects like improved nutrition, exercise training, cognitive behavioral therapy, and a variety of pharmacotherapies. ^7^ Bariatric surgery may be considered when conservative approaches fail; it is recommended for individuals with a body mass index (BMI) > 35 kg/m ^2^ with serious co-morbidities related to obesity. ^8^ A surgical procedure complements but does not replace behavioral, medical, and lifestyle treatments. ^7^ Management and treatment of obesity should have broader objectives than just the desired weight loss and should include risk reduction and health improvements. ^7^

One repeatedly stated challenge during weight loss is the undesired decrease in fat-free mass (FFM), such as muscle mass and bone mineral density (BMD). ^9^ This undesirable weight loss can have serious consequences for patients. Recent studies, for example, have revealed that patients who undergo bariatric surgery typically develop a pattern of osteoporosis characterized by bone loss, and they are therefore at greater risk of fractures than obese subjects or non-obese controls. ^10^ FFM is an important factor in basal metabolic rate, the regulation of body temperature, preservation of skeletal integrity, functional capacity, and quality of life. ^11^ Because of this, preserving FFM or minimizing its loss while losing fat mass is considered optimal and has been referred to as “high-quality weight loss”. ^12^

The literature reveals that after an excessive diet-induced weight loss program (≥20% of body weight), 27.8% of the weight lost consists of FFM if that program was carried out without additional therapy. ^11^ The same problem occurs with surgically-induced weight loss. After gastric bypass surgery with no other interventions, FFM accounts for 31.3% of the weight lost. ^11^

More recent literature shows the importance of resistance training and/or high-impact training and an intake of calcium and vitamin D to maintain or reduce FFM loss and, more specifically, the loss of BMD. ^13^ Both endurance- and resistance-type exercises seem to help preserve muscle mass during weight loss. ^9^ Additionally, resistance-type exercise improves muscle strength. ^9^ Inadequate protein intake results in a loss of FFM; thus, sufficient protein intake is highly recommended. ^9^

A recent survey in England revealed that some healthcare professionals caring for bariatric surgery patients did not follow recommendations on multivitamin, calcium, and vitamin D supplementation. ^14^ Furthermore, there is evidence that 67% of bariatric surgery patients are not physically active enough to maintain their weight loss (compared to 38% in the non-surgical group). ^15^ Considering these findings, it seems evident that the roles of exercise training and dietary supplements such as protein, calcium, and vitamin D during weight loss need further investigation, and their beneficial effects should be summarized to underline their importance.

Even though there is a well-established body of literature on exercise and dietary supplementation with protein, calcium, or vitamin D during weight loss, to the best of our knowledge, there have been no systematic reviews and meta-analyses to evaluate these interventions’ effects on preserving FFM. This systematic review and meta-analysis aimed to summarize current evidence on the maintenance of FFM through exercise training and/or dietary supplementation with protein, calcium, and vitamin D during weight loss interventions for adults. We aimed to calculate each individual intervention’s effects on the preservation of FFM during weight loss, namely exercise, protein supplementation, calcium supplementation, and vitamin D supplementation. We also investigated whether the combination of all four interventions (overall effect of exercise training and protein, calcium, and vitamin D supplementation) had a more beneficial impact on the maintenance of FFM than did each intervention individually. This led to the following research question:

What effect does exercise training have, with or without dietary supplementation (protein or calcium or vitamin D), on the preservation of FFM (BMD and muscle mass) among obese adults who have experienced weight loss (whether operative or conservative)?

We hypothesized that a) exercise training, with or without dietary supplementation, had a beneficial effect on maintaining FFM during weight loss, and b) that the combination of exercise therapy and dietary supplementation had a greater effect on maintaining FFM than did each intervention alone. We performed a systematic review involving a pairwise meta-analysis and an exploratory network meta-analysis to test our hypothesis.

## Methods

### Design

A systematic literature review involving a meta-analysis and a network meta-analysis was conducted in accordance with the PRISMA Extension Statement for Reporting of Systematic Reviews Incorporating Network Meta-analyses of Health Care Interventions. ^16^ The study protocol was registered on PROSPERO (registration number: CRD42019134651).

### Eligibility criteria

We included studies assessing overweight or obese (BMI of 25–29.9 kg/m ^2^ or BMI ≥ 30 kg/m ^2^)17 adults (≥ 18 years of age) undergoing diet- or surgery-induced weight loss and without a secondary diagnosis limiting their exercise activity (e.g., fractures, cancer, neurological diseases). Considered were randomized controlled trials or clinical trials comparing exercise training (defined as “at least 150–300 minutes of moderate-intensity aerobic physical activity; or at least 75–150 minutes of vigorous intensity aerobic physical activity; or an equivalent combination of moderate- and vigorous-intensity activity throughout the week” (p. 2), or as “muscle strengthening activities at moderate or greater intensity that involve all major muscle groups on 2 or more days a week” (p. 2) according to the WHO ^17^) alone or in combination with dietary supplementation (protein, calcium and/or vitamin D) with a placebo intervention, controlled comparison intervention or standard care. Studies assessing subjects’ FFM and/or BMD and/or muscle mass pre- and post-intervention were also included. Only studies in English, German, and French were included. Studies that used alternative treatment methods for weight loss (such as drugs) were excluded.

### Information sources

A systematic literature search was performed in the following electronic databases:

• Ovid Medline (date of inception [1946] to August 27, 2020) (RRID: SCR_002185)
• Ovid Embase (date of inception [1974] to August 27, 2020) (RRID: SCR_001650)
• Cochrane Central Register of Controlled Trials (CENTRAL) (date of inception [1996] to August 27, 2020) (RRID: SCR_001650)
• ISI Web of Science (date of inception [1900] to August 27, 2020)

### Search strategy

A search strategy was built using the following keywords: (“weight loss” OR “overweight” OR “obesity” OR “adiposity” OR “body weight changes”) AND (“physical training” OR “physical activity” OR “exercise” OR “exercise therapy”) AND (“dietary supplements” OR “nutritional” OR “supplementation” OR “protein” OR “amino acids” OR “calcium” OR “vitamin D”) AND (“body composition” OR “fat free mass” OR “lean mass” OR “bone density” OR “muscle mass”). Keywords and medical subject headings were identified with the assistance of a librarian from Bern University of Applied Sciences. Cochrane’s highly sensitive filter was used to identify randomized controlled trials. The search strategy was adapted for each database. Our detailed search strategy for the Ovid MEDLINE database can be found in Appendix A. ^70^ Additionally, the bibliographies of the relevant review articles and studies found via this search were examined for further potential studies. All the database searches were conducted on October 10, 2019, and again on August 27, 2020.

### Selection process

Two investigators (AR and CS) independently screened all the titles and abstracts of the publications revealed in the electronic databases. In cases of disagreement about an article’s inclusion, they discussed it until a consensus was found. The studies selected for inclusion were imported into EndNote X9.3.2.3 reference management software (Clarivate Analytics, Philadelphia, US) (RRID: SCR_014001), and duplicates were removed. An eligibility assessment was performed based on the title and abstract. To be included, the studies had to meet all the inclusion criteria. In cases of uncertainty regarding the article’s content based on its title and abstract, the full text was accessed and evaluated. The online Covidence platform was used to simplify the screening process. Full-text versions of all the studies meeting our inclusion criteria were retrieved for methodological quality assessment and data extraction.

### Data extraction

The information from each study included in the review was extracted and entered into an Excel file by the investigator. Data were extracted on study characteristics (e.g., author, year, country, study design, inclusion and exclusion criteria, funding, intervention groups, follow-up time, limitations), participants’ traits (e.g., sample size in each group, mean age, sex, mean weight, BMI and FFM, muscle mass, and BMD at baseline), and study results (outcome data, measurement methods, drop-outs). Missing data from four studies ^18^ ^–^ ^21^ were obtained by contacting their authors. If available, change score means and standard deviations (SD) were extracted. Otherwise, final values were used. SDs were derived from the 95% confidence intervals (95%CI) for two studies. ^22^ ^,^ ^23^ The SDs for seven studies ^19^ ^,^ ^21^ ^,^ ^24^ ^–^ ^28^ were imputed using the *p*-value. To obtain equal scales, outcome data reported in percentages were proportionally converted into kilograms. ^29^ ^–^ ^33^

### Statistical analysis

If only one study was available for a treatment comparison (i.e., statistical pooling was impossible), findings were reported as standardized mean differences (SMD) with their corresponding 95%CI. The minimum number of studies needed to perform a meta-analysis was set to 2 studies, if they were sufficiently similar, as recommended by Valentine *et al.* ^34^ and Higgins *et al.* ^35^ The analyses were performed using change scores, if possible, otherwise final values were used. ^36^ Where enough studies were available per treatment comparison and outcome, and the assumption of transitivity was fulfilled, a network meta-analysis was performed using a frequentist model. The assumption of transitivity was assessed for every study included in the network meta-analysis. ^37^ Studies had to be similar regarding their clinical and methodological aspects, with the exception of compared interventions.

A random effect model was chosen for all the meta-analyses because of the clinical and methodological diversity among the studies included. Pairwise meta-analyses were performed using the Meta statistical analysis package in R software (R Core Team, Austria) (RRID: SCR_00195). ^38^ The Netmeta package ^39^ was used for the network meta-analysis. SMDs were calculated and expressed as Hedges’ g. The DerSimonian–Laird estimator was used to analyze between-study variance (τ ^2^). ^40^ The Hartung–Knapp–Sidik–Jonkman adjustment for random effects models was also applied. ^41^ A meta-regression for the variables of age at baseline and BMI at baseline was calculated using a mixed-effects model. ^42^

All the outcomes of interest were reported as continuous data. The interpretation of effect sizes was made according to the Cochrane Handbook. ^43^ A small effect size was considered as 0.2 to 0.49, a moderate effect size as 0.5 to 0.79, and a large effect size as ≥ 0.8.

The SMD was selected as the effect size for the meta-analyses because the SMD enables a quick interpretation of the size of the effect. Interpreting a reduction in FFM or a change in BMD is not straightforward, and we believe that results are more clinically interpretable using SMDs. In addition, Takeshima *et al*. ^44^ demonstrated that the SMD is more generalizable than the MD.

Statistical heterogeneity between studies was assessed using a Chi ^2^ test and I ^2^ statistics. Those calculations were also interpreted according to the Cochrane handbook. ^43^ Results with a *p*-value < 0.05 were considered statistically significant. If studies assessed different groups, only data on the groups meeting our eligibility criteria were analyzed.

### Risk of bias assessment

To assess the quality of the studies selected, we used the revised Cochrane risk-of-bias tool for randomized trials (RoB 2.0), the updated version of the most-used tool for assessing the risk of bias in randomized trials. ^45^ Each criterion was evaluated according to the tool’s key questions and finally classified as “low risk”, “some concerns”, or “high risk”. The risk of bias assessment was performed after the data was extracted by the two reviewers independently (AR and CS). Disagreements between reviewers were resolved by discussion until a consensus was found. Potential publication bias could not be assessed using funnel plots or statistical tests, such as Egger’s test, because these methods do not possess enough power to distinguish chance from real asymmetry when fewer than 10 studies are involved in a pairwise meta-analysis. ^43^

Recommendations from the GRADE working group were used to rate the quality of the available evidence. ^46^

## Results

**Figure 1.**
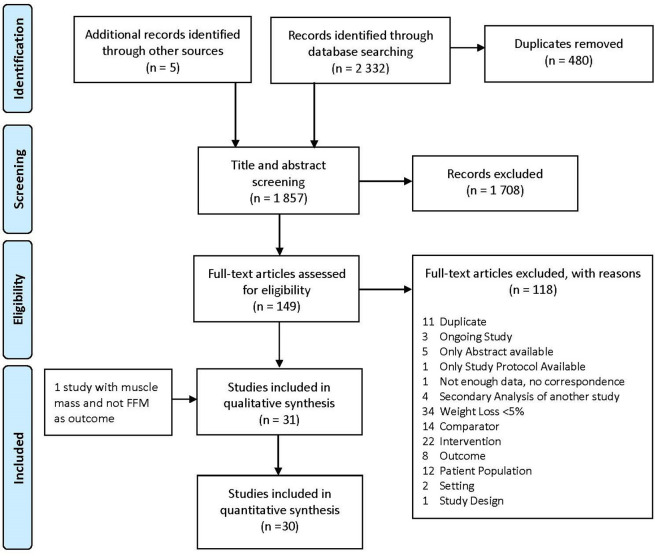
PRISMA flow chart describing the article selection process.

We found 31 eligible studies, but a quantitative synthesis was only possible for 30 of them. One study only reported muscle mass and not FFM as its outcome and, therefore, could not be included in comparisons with the others. ^47^ The study selection process is summarized in Figure 1. A list of all the included studies and a table of their individual characteristics are presented in Appendices B and C. All the studies were randomized controlled studies and were published between 1999 and 2019 with sample sizes ranging from 5 to 169 subjects. Participants’ ages ranged from 21 to 74 years, and BMIs ranged from 25.8 to 56.8 kg/m ^2^. Follow-up periods ranged from 4 weeks to 24 months. Most of the trials were from the USA (k = 11) ^19^ ^,^ ^20^ ^,^ ^22^ ^,^ ^24^ ^,^ ^30^ ^,^ ^32^ ^,^ ^33^ ^,^ ^48^ ^–^ ^51^ followed by Canada (k = 5) ^25^ ^,^ ^52^ ^–^ ^55^ and Brazil (k = 3). ^18^ ^,^ ^21^ ^,^ ^26^ Six studies used resistance training for their exercise training intervention ^25^ ^,^ ^32^ ^,^ ^47^ ^,^ ^55^ ^–^ ^57^ eight used aerobic training ^12^ ^,^ ^23^ ^,^ ^33^ ^,^ ^48^ ^,^ ^51^ ^,^ ^58^ ^–^ ^60^ and 17 used combined training programs. ^18^ ^–^ ^22^ ^,^ ^24^ ^,^ ^26^ ^–^ ^31^ ^,^ ^49^ ^,^ ^50^ ^,^ ^52^ ^–^ ^54^ All exercises interventions are described in Appendix C. Among the group of exercise training alone, different training modalities were used. Some had strength and other endurance training and among those who had the strength training different training parameters were chosen (i.e. different training volumes and intensities).

### Risk of bias assessment

**Figure 2.**
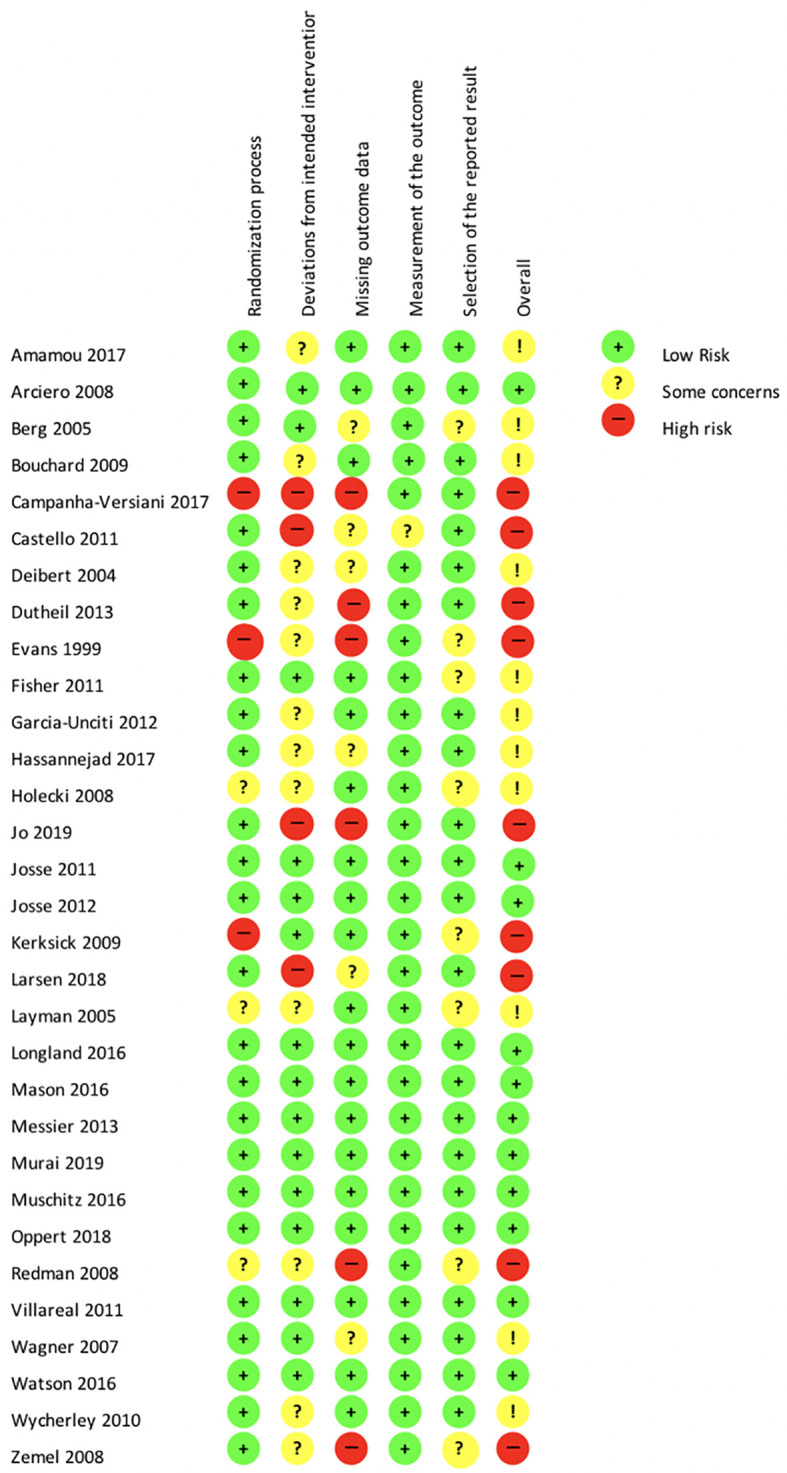
Risk of bias according to the revised Cochrane risk-of-bias tool for randomized trials (RoB 2.0). NB. “!” in the “overall” category corresponds to “Some concerns”.

Figure 2 presents the detailed results of the risk of bias assessment. The randomization process was clearly described in 73.3% of the studies. Deviations from the intended interventions were either not clearly described or inappropriately analyzed in 53.3% of the studies. Missing outcome data were reported properly in 56.7% of the studies. The measurement of the outcome data was reliable and valid in 96.7% of the studies, but 20% of them were at risk of a potential selective reporting bias. Fifty percent of the studies included in the network meta-analysis were conducted without mentioning sponsors or funding resources.

### FFM

***Effects of diet-induced weight loss on FFM***

We made 23 pairwise comparisons involving a total of 1642 patients to assess effects on the FFM outcomes. All the participants underwent a diet-induced weight loss program. The commonest comparison was Exercise Training versus a Control (k = 7), ^20^ ^,^ ^25^ ^,^ ^31^ ^–^ ^33^ ^,^ ^48^ ^,^ ^57^ followed by Exercise Training + High Protein versus Exercise Training (k = 6), ^12^ ^,^ ^22^ ^,^ ^52^ ^,^ ^54^ ^,^ ^56^ ^,^ ^59^ and Exercise Training + High Protein versus High Protein (k = 5). ^24^ ^,^ ^30^ ^,^ ^55^ ^,^ ^58^ ^,^ ^60^ “High Protein” meant that the participants exceeded the regular recommendation of 0.8g/kg body weight per day or hit 20% or more of caloric intake from protein. ^61^

**Figure 3.**
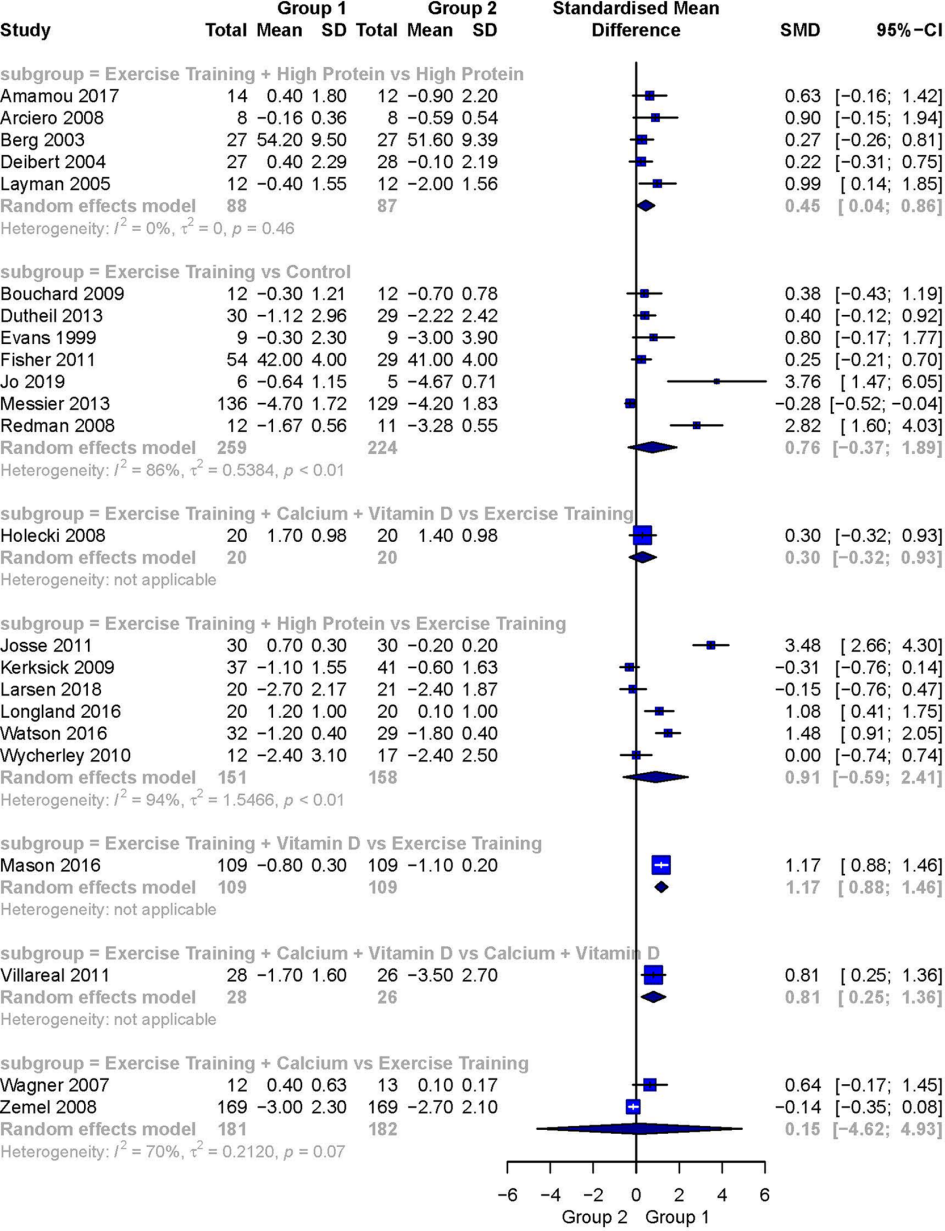
Forest plot of the head-to-head comparisons for the fat-free mass (kg) outcome during diet-induced weight loss. Data are presented as SMDs with 95%CIs. The FFM outcomes are expressed as change scores and final values.

Figure 3 presents a forest plot of the pairwise meta-analyses. The comparison of Exercise Training + High Protein versus High Protein ^24^ ^,^ ^30^ ^,^ ^55^ ^,^ ^58^ ^,^ ^60^ was the only statistically significant analysis including more than one study. It showed a small-to-moderately weighted effect size favoring Exercise Training + High Protein (SMD 0.45; 95%CI 0.04 to 0.86). It was also the only meta-analysis demonstrating no heterogeneity (I ^2^ = 0%). The comparison of Exercise Training versus a Control ^20^ ^,^ ^25^ ^,^ ^31^ ^–^ ^33^ ^,^ ^48^ ^,^ ^57^ showed a moderate but not statistically significant weighted effect size favoring the intervention group (SMD 0.76; 95%CI -0.37 to 1.89). The comparison of Exercise Training + High Protein versus Exercise Training ^12^ ^,^ ^22^ ^,^ ^52^ ^,^ ^54^ ^,^ ^56^ ^,^ ^59^ resulted in a large, but again, not statistically significant weighted effect size favoring the intervention group (SMD 0.91; 95%CI -0.59 to 2.41). The between-study heterogeneity for these two comparisons was large and statistically significant (I ^2^ = 84% and I ^2^ = 94%, respectively). The subgroup of Exercise Training + Calcium versus Exercise Training ^19^ ^,^ ^51^ showed a small effect size, with a wide 95%CI, favoring Exercise Training + Calcium (SMD 0.15; 95%CI -4.62 to 4.93). The heterogeneity for this comparison was large (I ^2^ = 70%). For the comparison of Exercise Training + Calcium + Vitamin D versus Exercise Training ^28^ a small but not statistically significant weighted effect size was detected favoring Exercise Training + Calcium + Vitamin D (SMD 0.30, 95%CI -0.32 to 0.93). Heterogeneity was not applicable. In the comparison of Exercise Training + Calcium + Vitamin D versus Calcium + Vitamin D ^62^ a large and statistically significant weighted effect size favoring Exercise Training + Calcium + Vitamin D was detected (SMD 0.81, 95%CI 0.25 to 1.36). Heterogeneity was not applicable.

The comparison of Exercise Training + Vitamin D versus Exercise Training ^49^ showed a large, statistically significant effect size (SMD 1.17; 95%CI 0.88 to 1.46) favoring Exercise Training + Vitamin D. Again, heterogeneity was not applicable.

**Figure 4.**
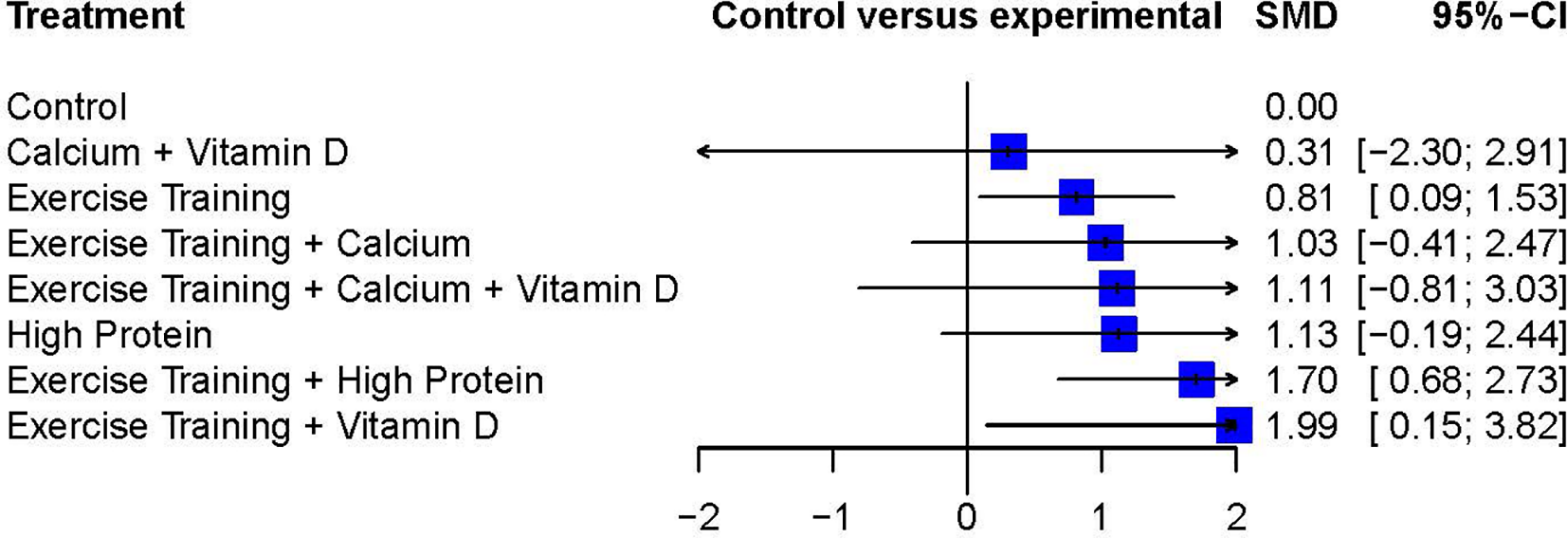
Network meta-analysis ranking and summary of weighted effect sizes.

**Figure 5.**
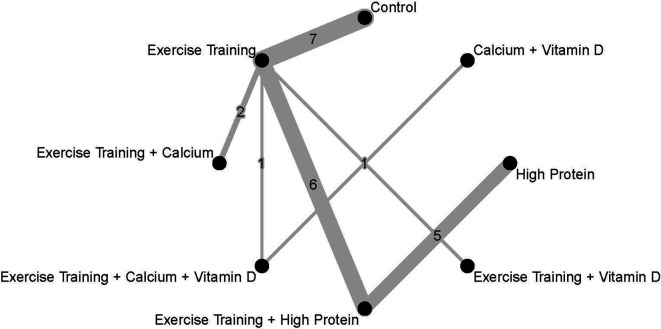
Network geometry and number of studies in each comparison. Every intervention was compared to a Control. Weighted effect sizes are presented as SMDs and their corresponding 95%CI.

In addition to the pairwise meta-analysis, a network meta-analysis assessed FFM outcomes after diet-induced weight loss. The Exercise Training + Vitamin D treatment resulted in the greatest weighted effect size (SMD 1.99; 95%CI 0.15 to 3.82) and therefore was ranked as the most effective treatment according to this network meta-analysis, followed by Exercise Training + High Protein (SMD 1.70; 95%CI 0.68 to 2.73) and High Protein alone (SMD 1.13; 95%CI -0.19 to 2.44). Three interventions showed statistically significant weighted effect sizes, and all of them included the Exercise Training treatment: Exercise Training + Vitamin D, Exercise Training + High Protein, and Exercise Training alone. The Calcium + Vitamin D treatment resulted in a relatively-small weighted effect size with a wide 95%CI (SMD 0.31, 95%CI -2.30 to 2.91) compared to other interventions. Figure 4 presents each treatment’s effect sizes compared to the control group as well as their ranking. The geometry of the network comprised n = 8 nodes and n = 7 edges. The network did not comprise any closed loops (i.e., parts of the network where all comparisons are connected to each other ^63^). It was, therefore, impossible to explore the inconsistency within the network by comparing direct and indirect treatment estimates, as suggested by Veroniki *et al.* ^64^ The network graph with the number of trials is presented in Figure 5. The pooled effect estimations of all the direct and network meta-analysis comparisons and their *p*-values are also presented in Appendices D and E.

A meta-regression was only applicable for comparing Exercise Training versus Controls for the variables of age and BMI at baseline. In the overall model, the age at baseline variable only explained 15.21% of the variability in the effect sizes and was not statistically significant (R ^2^: 39.41%, *p*-value: 0.33). There was only a weak relationship between the explanatory variable and the effect estimate (b1: -0.04; 95%CI -0.15 to 0.07, t: -1.11, *p*-value: 0.33). The variable of BMI at baseline explained 0.00% of the variability of the effect sizes in the overall model and was not statistically significant (R ^2^: 0.00%, *p*-value: 0.59). This explanatory variable could not be used as a predictor of the effect estimate (b1: 0.1; 95%CI **-**0.37 to 0.57, t: 0.57, *p*-value: 0.59).

***Effect of surgery-induced weight loss on FFM***

**Figure 6.**
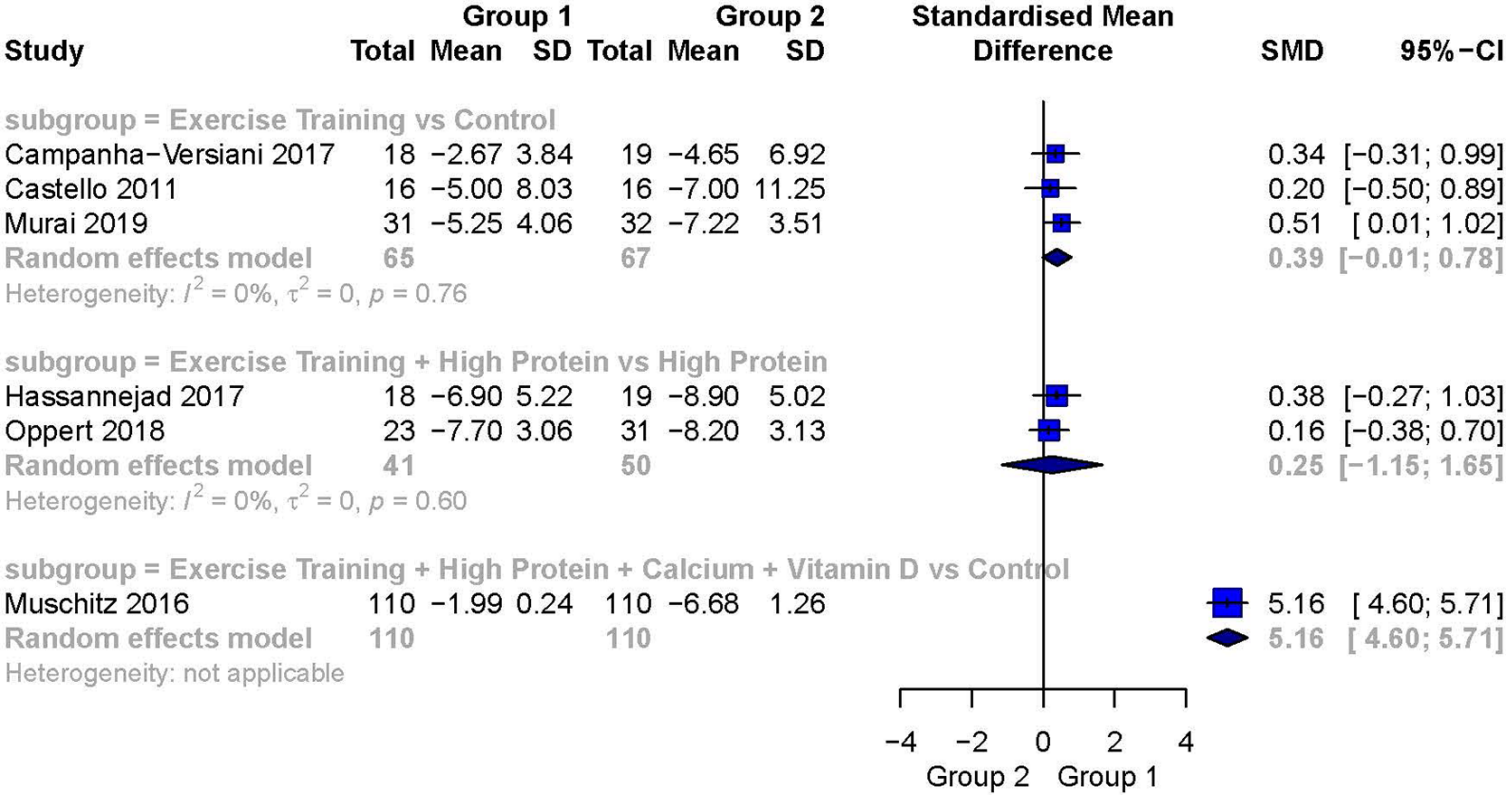
Forest plot of the meta-analysis of outcome change scores for fat-free mass (kg) during surgery-induced weight loss.

Six studies ^18^ ^,^ ^21^ ^,^ ^23^ ^,^ ^26^ ^,^ ^27^ ^,^ ^29^ including a total of 443 participants, reported change scores for FFM during surgery-induced weight loss. Figure 6 presents a summary forest plot of these results.

**Exercise training versus a control**

Three studies reported FFM as the outcome variable in this subgroup. ^18^ ^,^ ^21^ ^,^ ^26^ The analysis for this outcome showed a small to moderate weighted effect size in favor of Exercise Training over a Control (SMD 0.39; 95%CI -1.01 to 0.78), but the analysis was not statistically significant. There was no evidence of heterogeneity between these studies (I ^2^ = 0%).

**Exercise training + high protein versus high protein**

Two studies in this subgroup reported on FFM. ^23^ ^,^ ^27^ The analysis for this outcome showed a small weighted effect size favoring Exercise Training + High Protein over High Protein (SMD 0.25; 95%CI -1.15 to 1.65), but the result was not statistically significant. There was no evidence of heterogeneity between these studies (I ^2^ = 0%).

**Exercise training + high protein + calcium + vitamin D versus a control**

Only one study in this subgroup reported on FFM. ^29^ We detected a very large, statistically significant weighted effect size favoring Exercise Training + High Protein + Calcium + Vitamin D over the control group (SMD 5.16; 95%CI 4.60 to 5.71).

### BMD

***Effect of diet-induced weight loss on BMD***

Only one study investigated BMD during diet-induced weight loss. ^53^ The intervention group lost less total-body BMD than the control group. The comparison of Exercise Training + High Protein versus Exercise Training ^53^ showed a large weighted effect size (SMD 4.17; 95%CI 3.24 to 5.09) favoring Exercise Training + High Protein.

***Effect of surgery-induced weight loss on FFM***

**Figure 7.**
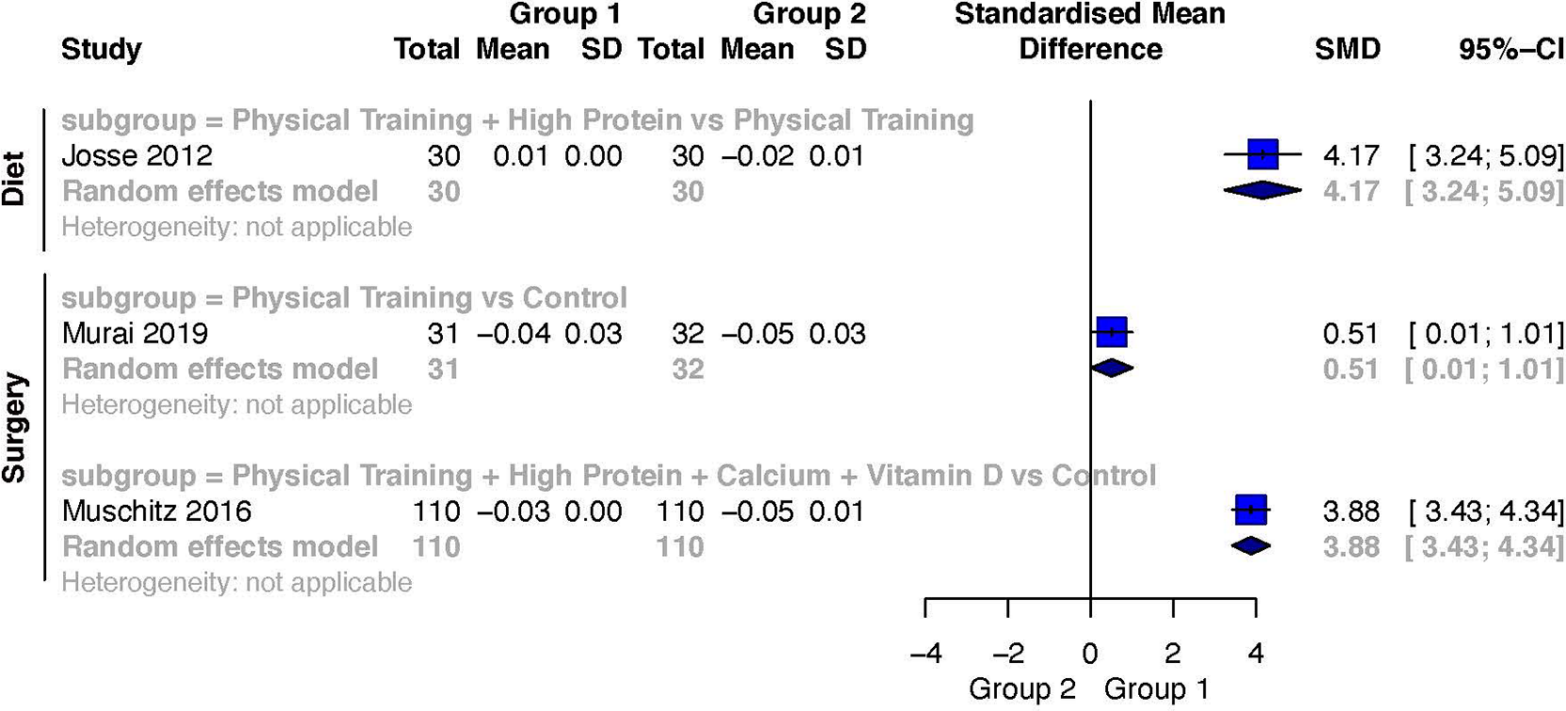
Weighted effects and their corresponding 95%CIs for the outcome change scores for total-body bone mineral density during diet- and surgery-induced weight loss.

Two studies investigated BMD after surgery-induced weight loss. ^18^ ^,^ ^29^ The intervention group lost less total-body BMD than the control group. The comparison of Exercise Training versus a Control ^18^ resulted in a moderate weighted effect size (SMD 0.51; 95%CI 0.01 to 1.01) favoring Exercise Training. Furthermore, the comparison of Exercise Training + High Protein + Calcium + Vitamin D versus a Control ^29^ also resulted in a large weighted effect size (SMD 3.88; 95%CI 3.43 to 4.34). A forest plot of the results for BMD is presented in Figure 7.

### Muscle mass

One four-armed study, including 25 participants, assessed muscle mass loss during diet-induced weight loss by comparing Exercise Training + High Protein versus a Control versus Exercise Training versus High Protein. ^47^ No statistically significant differences in muscle mass loss were reported between any of the groups. Nevertheless, the Exercise Training + High Protein group demonstrated the lowest muscle mass loss.

### Grade level of evidence

The level of evidence for each analysis is presented in Appendix F. The quality of evidence for diet-induced weight loss’s effects on FFM ranged from very low to moderate. The subgroup-analyses demonstrating a moderate level of evidence were: i) Exercise Training + High Protein versus Exercise Training and ii) Exercise Training + Calcium + Vitamin D versus Calcium + Vitamin D. The quality of evidence for surgery-induced weight loss’s effects on FFM also ranged from very low and moderate (Exercise Training + High Protein + Calcium + Vitamin D versus Vitamin D).

## Discussion

This study aimed to determine the effects of exercise training and protein, calcium, and vitamin D supplementation on the preservation of FFM during induced weight loss among overweight and obese adults. It also investigated whether the combination of all these interventions (the overall effect of exercise training and protein, calcium, and vitamin D supplementation) had a more beneficial impact on the maintenance of FFM than each intervention alone. This was done via a systematic review of the literature that found 31 randomized controlled trials covering these topics. Data on the 2560 participants in those trials was investigated using pairwise and network meta-analyses, and the trials are presented in Appendix B. ^71^ In accordance with our hypothesis, results underlined the importance of exercise training and sufficient protein intake when seeking to preserve FFM during weight loss in obese adults. The effects of calcium and vitamin D supplementation remain controversial, and further research is needed.

Regarding diet-induced weight loss’s effects on FFM, this study’s results indicated that Exercise Training plus dietary supplementation was superior to Exercise Training alone, to dietary supplementation alone, and to no interventional therapy during weight loss. The results of our pairwise meta-analysis showed that the Exercise Training + High Protein intervention was superior in every comparison and independent of the outcome and type of induced weight loss. Previous research reported similar findings. ^9^

Nevertheless, there was heterogeneity in the results of studies comparing Exercise Training + High Protein versus Exercise Training during diet-induced weight loss. This heterogeneity could be partially due to quality differences in the studies. The two studies which favored the Exercise Training + High Protein group in the preservation of FFM ^54^ ^,^ ^59^ were rated as “low risk” for bias, whereas the three studies claiming the contrary were “high risk” for bias or, at the very least, showed “some concerns”. ^12^ ^,^ ^22^ ^,^ ^56^

Results consistently favored exercise training over the control intervention during diet-induced weight loss, although this was not always statistically significant. These findings were in line with previous reviews. ^9^ ^,^ ^11^ ^,^ ^15^ One included study ^20^ stood out for favoring the Control over Exercise Training; it reported the change score for FFM in kg. However, when considering FFM loss in relation to the overall amount of weight lost, the Exercise Training group lost more than the control group. ^20^ However, only reporting the FFM change score in kg may lead to a misinterpretation of a study’s results. Future studies should therefore report both endpoints, namely the change score for FFM in kg as well as the FFM loss in relation to the overall amount of weight loss.

Regarding the results of our network meta-analysis, the Exercise Training + vitamin D intervention had the largest weighted effect size on FFM during diet-induced weight loss, followed by the Exercise Training + High Protein intervention *.* It should be mentioned that the weighted effect size calculation for Exercise Training + Vitamin D was based on a single study. Researchers and clinicians should therefore be careful interpreting these results.

Regarding the effects of surgery-induced weight loss on FFM, the studies showed a tendency to favor exercise training over controls in our pairwise meta-analysis, but these effects were not statistically significant. Further studies are needed to investigate the effects of post-bariatric surgery exercise training on bone and muscle mass and outcomes assessing the exercise function of the participants.

The combination of exercise training and high protein, calcium, and vitamin D supplementation seems to be the most effective treatment for maintaining FFM during surgery-induced weight loss. However, only one relevant study investigating this combination of interventions could be found, which limits its informative value.

After our analyses, a new controlled trial was published investigating the effects of exercise and protein, calcium, and vitamin D supplementation during weight loss. ^65^ The authors concluded that calcium and vitamin D appeared to provide no additional benefits to dietary and exercise interventions in terms of body composition during weight loss. However, the same researchers discussed the possible beneficial effects of calcium and vitamin D supplementation for persons who were deficient in these micronutrients before supplementation. This might thus limit the number of people who could benefit from calcium and vitamin D supplementation. Yet it might also explain why our review found such a large effect during surgery-induced weight loss since bariatric surgery can result in poor absorption and limited nutritional intake. ^9^ ^,^ ^11^ ^,^ ^13^ Surgery-induced weight loss is more likely to cause nutrient deficiencies that are important for FFM (including calcium, vitamin D, and protein ^10^) than is dietary-induced weight loss. In a meta-analysis by Krieger *et al*. , ^66^ a higher daily protein intake of > 1.05 to ≤ 1.20 g/kg of body weight was associated with greater FFM maintenance than a lower protein intake of < 0.7 g/kg of body weight during weight loss. Thus, the frequently recommended daily protein intake of 0.8 g/kg of body weight may be inadequate for individuals during weight loss. ^66^ The meta-analysis by Stockton *et al.* ^67^ reported that vitamin D supplementation improved muscle function in adults with a vitamin D deficiency but not in non-deficient individuals. Another meta-analysis found a small overall beneficial effect of vitamin D supplementation on BMD at the femoral neck, with larger positive effects in individuals with 25-hydroxyvitamin D levels ≤ 20 nmol/L. ^68^ Goode *et al.* ^69^ observed dramatically lower intestinal calcium uptake and elevated bone resorption markers among patients who had undergone a gastric bypass, even with the recommended calcium (1.2 g/d) and vitamin D (8 μg/day) intake. Those authors concluded that individuals who underwent bariatric surgery with a malabsorptive component may require even higher dosages to avoid bone loss. However, more research is needed to investigate this question: if individuals undergoing surgery-induced weight loss benefit from calcium and vitamin D supplementation, then why is this not true for non-deficient individuals undergoing diet-induced weight loss?

The methodologies chosen for estimating FFM might also influence FFM values. One review reported that dual-energy X-ray absorptiometry (DXA) was the most popular method used but that it also has certain biases that may lead to FFM overestimations. ^70^ As almost all of the included studies (23 out of 29) used DXA to measure FFM, we might have an overestimation, but it is unlikely that the method used explains the major differences. Only one study used skinfold measurements to estimate FFM. ^26^ This method relies on the tester’s technique and skill and does not measure FFM *per se*; rather, it provides data for calculations to predict FFM based upon body density and fat percentage. ^70^ Three studies assessed FFM using bioelectrical impedance analysis (BIA). ^19^ ^,^ ^27^ ^,^ ^28^ As this method has inherently large predictive errors, it is insensitive to small improvements in response to treatment. ^70^ Therefore, studies assessing FFM using BIA might have missed small changes in FFM. There is no one-size-fits-all approach for the assessment of FFM in obese subjects, but future research should be aware of each modality’s benefits and drawbacks and choose those most appropriate to their situation. ^70^

Our work’s major strengths are the large number of studies included (k = 31) and the large sample size (n = 2560). The quality assessment performed by two reviewers independently is a further strength. Combining a wide variety of treatments and merging diet- and surgery-induced weight loss strategies led to a broad overview and added new knowledge to this field of research. However, the study also had some limitations. The first concerned our search strategy. Indeed, few synonyms were used for the secondary outcomes; thus, some potentially eligible studies may have been missed. Another limitation was that 12 of the 31 studies included only evaluated women. It could thus be difficult to generalize the review’s findings to a mixed or exclusively male population. In addition, the studies included heterogeneous samples (e.g., age range, BMI, and follow-up length) and a diversity of exercise training interventions and the supplemental dosages. This might also explain the significant heterogeneity in our meta-analyses, as might the sometimes very low number of participants in individual studies.

Another issue was that the quality of evidence—measured using the “grade” approach—ranged from very low to moderate quality. None of the studies was rated as having high-quality evidence. The true effects of the interventions examined might, therefore, differ substantially from the estimated effects presented.

Additionally, our network meta-analysis did not comprise closed loops (i.e., a set of treatments which have been compared against each other). Therefore, it was impossible to analyze our network’s internal consistency by comparing direct and indirect treatment estimates. ^64^ It should be noted that our network meta-analysis was exploratory in character and, therefore, should be interpreted with caution. A further statistical limitation was that we did not plan a meta-regression from the beginning; thus, we did not report it in the study protocol. It should be interpreted with skepticism as it included fewer than 10 studies. ^43^ However, it is of clinical importance that the variables of age and BMI at baseline seemed to have no influence on the treatment effects. A more conclusive result will require further investigation. The reasoning behind pooling data when only two studies are available could also be questioned, although this remains in line with current recommendations. ^34^ ^,^ ^35^ The fact that a network meta-analysis was carried out could also be criticized considering the small number of articles included. However, this method ensures that only comparable data are analyzed together.

Some studies only provided incomplete outcome data, which obliged us to calculate results as described in the Methods section. With respect to further empirical trials, separate research studies are needed to better identify how combining exercise training with protein, calcium, and vitamin D supplementation for obese or overweight patients during diet- or surgery-induced weight loss affects BMD and muscle mass independently. Additionally, the long-term effects and cost-effectiveness of exercise interventions and dietary supplementation for obese patients undergoing weight loss should be examined.

We should also mention that all the types of exercise reported in our review were classified as “exercise therapy”, with no distinctions made between strength training and endurance training, even though these do not have the same effects on the preservation of FFM. ^71^ Some of the chosen training modalities do not target an increase in muscle mass or a decrease in fat mass which might underestimate the effectiveness on the outcome FFM.

## Conclusion

The present systematic review, including a meta-analysis and exploratory network meta-analysis, investigated the effects of exercise training and protein, calcium, and vitamin D supplementation, alone and in every possible combination, on the preservation of fat-free mass (FFM) as overweight or obese adults undergo diet- or surgery-induced weight loss. Results showed consistently more positive outcomes for exercise training over control interventions as well as Exercise Training + High Protein over Exercise Training alone. These findings underlined the importance of exercise training and sufficient protein intake when seeking to preserve FFM during weight loss in overweight or obese adults, regardless of the weight loss approach used. The effects of calcium and vitamin D supplementation remain controversial. It has been hypothesized that only individuals deficient in these nutrients will benefit from such an intervention, and future research should investigate this. The gaps in knowledge regarding combining all these treatment interventions to maintain FFM during the weight loss undergone by overweight or obese adults have not yet been fully closed.

## Data availability

### Underlying data

Figshare: DATA SET - EFFECTS OF EXERCICE TRAINING AND DIETARY SUPPLEMENTATION ON FAT FREE MASS AND BONE MASS DENSITY DURING WEIGHT LOSS https://doi.org/10.6084/m9.figshare.17086520.

The project contains the following underlying data:

• [data_SR_Roth.xlsx] (Raw deidentified data).

### Extended data

Figshare: Appendix A Search Strategy Medline Ovid https://doi.org/10.6084/m9.figshare.17113475

This project contains the following extended data:

• AppendixA_Search_Strategy_Ovid.pdf

Fighare: Appendix B: List of all included studies https://doi.org/10.6084/m9.figshare.17113511

This project contains the following extended data:

• AppendixB_List_Included_Studies.pdf

Figshare: Appendix C: Characteristics of studies https://doi.org/10.6084/m9.figshare.17113520.v2.

This project contains the following extended data:

• AppendixC_Characteristics_of_Studies.pdf

Figshare: Appendix D: Netleague table https://doi.org/10.6084/m9.figshare.17113520

This project contains the following extended data:

• AppendixD_netleagueTable.csv

Figshare: Appendix E p-scores:

https://doi.org/10.6084/m9.figshare.17113586

This project contains the following extended data:

• AppendixE_PScore.xlsx

Figshare: Appendix F Grade

https://doi.org/10.6084/m9.figshare.20424585.v1

## Reporting guidelines

The Prisma checklist for this systematic review is available at: https://doi.org/10.6084/m9.figshare.17085932

Data are available under the terms of the Creative Commons Zero “No rights reserved” data waiver (CC0 1.0 Public domain dedication).
