# Supplementary material for: Effects of exercise training and dietary supplement on fat free mass and bone mass density during weight loss – a systematic review and meta-analysis
Source: F1000Res. 2022 Sep 6;11:8. Originally published 2022 Jan 5. [Version 3] doi: 10.12688/f1000research.75539.3 (PMC9396077; doi:10.12688/f1000research.75539.3)
Supplement: Peer review history

# Peer review: Reviewer response for version 2

**DOI:** 10.5256/f1000research.135721.r147413

**Published:** 2022-08-22

**Authors:** Nahid Ramezani-Jolfaie

**Affiliations:** 1 Department of Community Medicine, School of Medicine, Hormozgan University of Medical Sciences, Bandar Abbas, Iran

The revised manuscript has been improved and my concerns have been resolved. I have no more comments.

Are the rationale for, and objectives of, the Systematic Review clearly stated?

Yes

Is the statistical analysis and its interpretation appropriate?

Yes

Are sufficient details of the methods and analysis provided to allow replication by others?

Partly

Are the conclusions drawn adequately supported by the results presented in the review?

Yes

Reviewer Expertise:

Nutritionist & Diet therapist

I confirm that I have read this submission and believe that I have an appropriate level of expertise to confirm that it is of an acceptable scientific standard.

# Peer review: Reviewer response for version 2

**DOI:** 10.5256/f1000research.135721.r147412

**Published:** 2022-08-15

**Authors:** Stéphanie Saxer

**Affiliations:** 1 Department of Pulmonology, University Hospital Zurich, University of Zurich, Zurich, Switzerland; 2 Ostschweizer Fachhochschule, Departement Gesundheit, St. Gallen, Switzerland

The authors responded in detail to all the comments. Only two more points need to be considered:

- You stated that the title has been changed from ‘physical activity’ to ‘exercise training’, however, this cannot be seen.
- Furthermore, the definition you included for ‘exercise training’ includes the recommendations for physical activity in daily life. You should rewrite this sentence to ‘exercise training or being physically active’ followed by the definition and probably also make a link to Appendix C where the interventions are described.

Are the rationale for, and objectives of, the Systematic Review clearly stated?

Yes

Is the statistical analysis and its interpretation appropriate?

Yes

Are sufficient details of the methods and analysis provided to allow replication by others?

Yes

Are the conclusions drawn adequately supported by the results presented in the review?

Yes

Reviewer Expertise:

Exercise, physical activity

I confirm that I have read this submission and believe that I have an appropriate level of expertise to confirm that it is of an acceptable scientific standard.

# Response

**Published:** 2022-08-25

**Authors:** Chloé Schorderet

**Affiliations:** HES-SO Valais Wallis, Switzerland

Thank you for your comments. 

 We have now changed the title. The term "physical activity" has been replaced with "exercise training".

 We have also added the term "physically active" before the definition of "exercise training" and clarified that the types of exercise are described in Appendix C for each study.

# Peer review: Reviewer response for version 1

**DOI:** 10.5256/f1000research.79422.r127522

**Published:** 2022-06-13

**Authors:** Nahid Ramezani-Jolfaie

**Affiliations:** 1 Department of Community Medicine, School of Medicine, Hormozgan University of Medical Sciences, Bandar Abbas, Iran

In this systematic review and meta-analysis, Roth *et al.* examined the effect of exercise training, protein, calcium, and vitamin D supplementation on the preservation of fat-free mass during non-surgical and surgical weight loss and of the combination of all interventions together in adults with obesity. This manuscript deals with an interesting topic. However, there are several points to be addressed. The language needs editing. Furthermore, the process should be thoroughly described. Generally, the paper is well written and the topic is highly relevant. Nevertheless, there are some limitations in this piece of work that could be improved. 

- The method of the search strategy is faced with limitations. First, the search query is not designed properly, so it could not find all relevant papers, especially the papers that have reported “fat free mass” and “bone mass density” as secondary outcomes. Second, some important databases such as PubMed, Scopus, and Embase have been not searched.
- Statistical analysis: The authors stated, “To have equal scales, outcome data reported in percentages were proportionally converted into Kilograms”. So, why did the authors use the SMD? SMD is usually used when studies have reported the outcome variables using different scales and scales cannot be converted to each other.
- Eligibility criteria: Did you include also non-randomized studies? Regarding the inclusion of non-RCTs, I highly suggest that the authors include only RCTs or perform subgroup analysis based on randomization.
- The section of eligibility criteria lacks any description/definition of high protein. Please describe high protein thoroughly.
- From Figure 1, I do not understand what is meant by “inadequate patient population” and “inadequate setting”. Please rephrase this to improve clarity.
- Certainty of evidence assessment needs to be performed with the GRADE approach and findings to be included in the results section and conclusion section of the abstract.
- Please provide information regarding the gender of participants in the table of characteristics of studies.
- The manuscript must be also revised with regard to the English language.

Are the rationale for, and objectives of, the Systematic Review clearly stated?

Yes

Is the statistical analysis and its interpretation appropriate?

Yes

Are sufficient details of the methods and analysis provided to allow replication by others?

Partly

Are the conclusions drawn adequately supported by the results presented in the review?

Yes

Reviewer Expertise:

Nutritionist & Diet therapist

I confirm that I have read this submission and believe that I have an appropriate level of expertise to confirm that it is of an acceptable scientific standard, however I have significant reservations, as outlined above.

# Response

**Published:** 2022-08-03

**Authors:** Chloé Schorderet

**Affiliations:** HES-SO Valais Wallis, Switzerland

**Point-to-point** **response**

- **Comment 1:**

The method of the search strategy is faced with limitations. First, the search query is not designed properly, so it could not find all relevant papers, especially the papers that have reported “fat free mass” and “bone mass density” as secondary outcomes. Second, some important databases such as PubMed, Scopus, and Embase have been not searched.

**Authors' response:**

 Thank you for this comment. We agree that the search terms for the secondary outcomes were not exhaustive. However, within the manuscript, only an overview of the search terms is presented. We searched the databases with more details as presented in Appendix A. For example, the terms muscle and bone were combined with adjacency of 3 words with the following terms:

 #40 ((muscle* or bone*) adj3 {health* or mass* or volume* or strength* or density or lass or augment* or metabolism* or turn?over* or preservation*)).ti,ab,kw.

 However, we added this point in the limitations of the manuscript.

 Discussion, page 13:

*A first limitation concerns the search strategy. Indeed, few synonyms were used for the secondary outcomes.*

 Regarding the second part of the comment, we followed the recommendations of the Cochrane Handbook for Systematic Reviews of Interventions (https://training.cochrane.org/handbook/current/chapter-04). To clarify, we searched for eligible studies in Medline (using Ovid's search engine). Records were also searched in EMBASE (using Ovid's search engine). In addition, we searched also in Cochrane Central and Web of Science. We believe that the chosen databases fulfilled the requirements of the Cochrane Handbook for Systematic Reviews.

- **Comment 2:**
**  **

Statistical analysis: The authors stated, “To have equal scales, outcome data reported in percentages were proportionally converted into Kilograms”. So, why did the authors use the SMD? SMD is usually used when studies have reported the outcome variables using different scales and scales cannot be converted to each other.

**Authors' response:**

 The selection of the appropriate effect size is controverse discussed in the literature. Several guidelines present opposing recommendations. For example, the Cochrane Collaboration suggests using the MD if all studies used the same outcome measure [1]. In contrast, Borenstein [2] recommends the use of the SMD when the clinical interpretation of outcome measures is not familiar to all readers. The advantage of SMD as an effect size is that there are easily understood rules of thumb that allow for quick interpretation of the effect size. In our case, the interpretation of the reduction of fat-free mass or change in bone mass density is not straightforward and we believe that results are clinically more interpretable when SMDs are used. In addition, Takeshima *et al.* [3] demonstrated that the SMD is more generalizable than the MD. We adapted the manuscript to clarify this point.

 Methods, statistical analysis, page 6:

*The SMD was selected as effect size for the meta-analyses because they allow a quick interpretation of the size of the effect. The interpretation of the reduction of fat free mass or a change in bone mass density is not straightforward and we believe that results are clinically more interpretable when SMDs are used. In addition, Takeshima et al.* *[3]* *demonstrated that the SMD is more generalizable than the MD.*

- **Comment 3: **

Eligibility criteria: Did you include also non-randomized studies? Regarding the inclusion of non-RCTs, I highly suggest that the authors include only RCTs or perform subgroup analysis based on randomization.

**Authors' response:**

 Thank you for your comment. Yes, only RCTs were included in this systematic review. We specified this point in the results section.

 Results, page 7:

*All included studies were randomized controlled studies.*

- **Comment 4: **

The section of eligibility criteria lacks any description/definition of high protein. Please describe high protein thoroughly.

**Authors' response:**

 Thank you for your comment. The term high protein has now been defined in the manuscript.

 Results, FFM, effect of diet induced weight loss on FFM, page 8

*“High Protein” meant that the participants exceeded the regular recommendation of 0.8g/kg body weight per day or hit 20% or more of caloric intake from protein* *[4]* *.*

- **Comment 5:  **

From Figure 1, I do not understand what is meant by “inadequate patient population” and “inadequate setting”. Please rephrase this to improve clarity.

**Authors' response:**

 Thank you for your comment. This term is indeed imprecise. We have now removed it from the flow chart. The term "inadequate" meant that studies were not included because the comparator/intervention/outcome/patient population/setting/study design did not meet the inclusion criteria.

- **Comment 6:**

Certainty of evidence assessment needs to be performed with the GRADE approach and findings to be included in the results section and conclusion section of the abstract.

**Authors' response:**

 We agree with you that the certainty of evidence should be presented. We added the following sections to the manuscript:

 Methods, risk of bias assessment, page 7:

*Recommendations from the GRADE working group were used to rate the quality of the available evidence.*

 Results, grade level of evidence, page 12:

*The level of evidence for each analysis is presented in Appendix F. For diet induced weight loss on FFM the quality of evidence ranged from very low to moderate. The subgroup-analyses with a moderate level were: i) Exercise + High Protein vs. Exercise and ii) Exercise + Calcium + Vitamin D vs. Calcium + Vitamin D. For surgery induced weight loss on FFM the level of evidence ranged between very low and moderate (Exercise + High Protein + Calcium + Vitamin D vs. Vitamin D).*

 Discussion, page14:

*Another issue is that the quality of evidence measured using the "grade" approach ranged from very low to moderate quality. No analysis was rated as high-quality evidence. Therefore, the true effects might substantially differ from the presented estimated effects.*

- **Comment 7: **

Please provide information regarding the gender of participants in the table of characteristics of studies.

**Authors' response:**

 Thank you for your comment. We added the gender of participants in the table of characteristics of studies (Appendix C).

  

- **Comment 8:**

The manuscript must be also revised with regard to the English language.

**Authors' response:**

 Thank you for your comment. We used the services of a copy editor to improve the language.

**References**

 [1] Higgins JPT, Thomas J, Chandler J, Cumpston M, Li T, Page MJ, et al. Cochrane Handbook for Systematic Reviews of Interventions version 6.3 (updated February 2022), www.training.cochrane.org/handbook; 2022.

 [2] Borenstein M, Hedges LV, Higgins JP, Rothstein HR. Introduction to meta-analysis. John Wiley & Sons; 2021.

 [3] Takeshima N, Sozu T, Tajika A, Ogawa Y, Hayasaka Y, Furukawa TA. Which is more generalizable, powerful and interpretable in meta-analyses, mean difference or standardized mean difference? BMC medical research methodology 2014;14:1-7

 [4] World Health Organization. Protein and amino acid requirements in human nutrition, Report of a Joint WHO/FAO/UNU Expert Consultation. WHO Technical Report Series. World Health Organization ed. World Health Organization; 2007.

# Peer review: Reviewer response for version 1

**DOI:** 10.5256/f1000research.79422.r125759

**Published:** 2022-03-18

**Authors:** Stéphanie Saxer

**Affiliations:** 1 Department of Pulmonology, University Hospital Zurich, University of Zurich, Zurich, Switzerland; 2 Ostschweizer Fachhochschule, Departement Gesundheit, St. Gallen, Switzerland

The study of Roth et al. summarizes the effect of exercise training and dietary supplement on fat free mass and bone mass density during weight loss in adults with overweight or obesity.

 The study is well designed and reported according to PRISMA guidelines. The study is well written; however, some English corrections need to be done (e.g. was not statistically significant).

**Title:**

- Change physical activity to exercise training because the study is not about physical activity.

**Abstract:**

- Omit “The effect of calcium and vitamin D supplementation remains controversial and further research are needed.” Or add these results in the abstract.
- Add that overweight patients were also included.

**Introduction:**

- Line 7: correct: “… but also could be cost-effective.”

**Methods:**

- Eligibility criteria: line 4: two times exercise.
- Exercise training is a broad term, define it more clearly.

**Results:**

- Figure 1: please comment on the excluded studies more clearly, what is meant with “inadequate”.
- Figure 2: An explanation of the yellow “!” is missing.

**Discussion:**

- Last paragraph of the discussion: Please present more details about the effect of different types of exercise training on the preservation of the FFM. Probably your study showed no clear favor of exercise training alone because not the right type of exercise training and intensity was chosen.
- You included around 12 studies which were only performed in women. However, there is no statement about this fact. Are there any differences between men and women?

Are the rationale for, and objectives of, the Systematic Review clearly stated?

Yes

Is the statistical analysis and its interpretation appropriate?

Yes

Are sufficient details of the methods and analysis provided to allow replication by others?

Yes

Are the conclusions drawn adequately supported by the results presented in the review?

Yes

Reviewer Expertise:

exercise, physical activity

I confirm that I have read this submission and believe that I have an appropriate level of expertise to confirm that it is of an acceptable scientific standard.

# Response

**Published:** 2022-08-03

**Authors:** Chloé Schorderet

**Affiliations:** HES-SO Valais Wallis, Switzerland

**Point-to-point** **response**

- **Comment 1**:

Change physical activity to exercise training because the study is not about physical activity.

**Authors' response:**

 Thank you for your comment. We replaced the term "physical activity" with the term "exercise training" throughout in the title of the manuscript.

  

- **Comment 2**:

Omit “The effect of calcium and vitamin D supplementation remains controversial and     further research are needed.” Or add these results in the abstract.

**Authors' response:**

 Thank you for your comment. We omitted the sentence “The effect of calcium and vitamin D supplementation remains controversial and further research are needed”.

  

- **Comment 3:**

Add that overweight patients were also included.

**Authors' response:**

 Thank you for your comment. We included this information in the abstract.

 Abstract, page 2:

***Methods*** *: A systematic review was performed with a pairwise meta-analysis and an* *exploratory network meta-analysis according to the PRISMA statement. Studies* *assessing adults with overweight or obesity undergoing a weight loss and without* *secondary diagnosis limiting their exercise activity were included.*

  

- **Comment 4:**

Line 7: correct: “… but also could be cost-effective.”

**Authors' response:**

 Thank you for your comment. We modified the sentence.

 Introduction, page 4:

*The literature reports that weight loss within this range not only has a beneficial* *impact on several obesity-related health conditions and co-morbidities but can also* *be cost-effective.*

  

- **Comment 5: **

Eligibility criteria: line 4: two times exercise.

**Authors' response:**

 Thank you very much. We corrected this error of inattention.

  

- **Comment 6**:

Exercise training is a broad term, define it more clearly

**Authors' response:**

 Thank you for your comment. The exercise regimen has been defined according to the physical activity guidelines by the WHO [1]. The term exercise training, therefore, meant the performance of “at least 150–300 minutes of moderate-intensity aerobic physical activity; or at least 75–150 minutes of vigorous intensity aerobic physical activity; or an equivalent combination of moderate- and vigorous-intensity activity throughout the week” (p. 2) [1], or the performance of “muscle strengthening activities at moderate or greater intensity that involve all major muscle groups on 2 or more days a week” (p. 2) [1]. We adapted the manuscript in order to clarify this point.

 Method, eligibility criteria, pages 4 and 5:

*Considered were randomized controlled trials or clinical trials comparing exercise training (defined as “at least 150–300 minutes of moderate-intensity aerobic physical activity; or at least 75–150 minutes of vigorous intensity aerobic physical activity; or an equivalent combination of moderate- and vigorous-intensity activity throughout the week” (p. 2), or as “muscle strengthening activities at moderate or greater intensity that involve all major muscle groups on 2 or more days a week” (p. 2) according to the WHO* *[1]* *) alone or in combination with dietary supplementation (protein, calcium and/or vitamin D) with a placebo intervention, controlled comparison intervention or standard care.*

  

- **Comment 7: **

Figure 1: please comment on the excluded studies more clearly, what is meant with “inadequate”. -> can you expand on inclusion criteria for each “inadequate statement”

**Authors' response:**

 Thank you for your comment. This term is indeed imprecise. We have now removed it from the flow chart. The term "inadequate" meant that studies were not included because the comparator/intervention/outcome/patient population/setting/study design did not meet the inclusion criteria.

  

- **Comment 8:**

Figure 2: An explanation of the yellow “!” is missing.

**Authors' response:**

 Thank you for your comment. We added the meaning of the yellow “!” in the legend of Figure 2.

 Page 9

*Figure 2. Risk of bias according to the revised Cochrane risk-of-bias tool for randomized trials (RoB 2.0).* *NB. “!” in the “overall” category corresponds to "Some concerns".*

  

- **Comment 9**:

Last paragraph of the discussion: Please present more details about the effect of different types of exercise training on the preservation of the FFM. Probably your study showed no clear favor of exercise training alone because not the right type of exercise training and intensity was chosen.

**Authors' response:**

 Thank you for your comment. We added information in the results and in the discussion to clarify this point.

 Results, page 8

*All exercises interventions are described in Appendix C. Among the group of exercise training alone, different training modalities were used. Some had strength and other endurance training and among those who had the strength training different training parameters were chosen (i.e. different training volumes and intensities).*

 Discussion, page 16:

*We should also mention that all the types of exercise reported in our review were classified as “exercise therapy”, with no distinctions made between strength training and endurance training, even though these do not have the same effects on the preservation of FFM* *[2]* *. Some of the chosen training modalities do not target an increase in muscle mass or a decrease in fat mass which might underestimate the effectiveness on the outcome FFM.*

  

- **Comment 10: **

You included around 12 studies which were only performed in women. However, there is no statement about this fact. Are there any differences between men and women?

**Authors' response:**

 You are right this is important to consider. We added two sentences about this point in the discussion. In addition, we also added this variable in Appendix C.

 Discussion, page 13:

*Another limitation is that 12 of the 31 included studies evaluated only women. Therefore, it could be difficult to generalize the findings of this review to a mixed or to a male population.*

**References**

 [1] WHO guidelines on physical activity and sedentary behaviour. Geneva: World Health Organization; 2020.

 [2] Willis LH, Slentz CA, Bateman LA, Shields AT, Piner LW, Bales CW, et al. Effects of aerobic and/or resistance training on body mass and fat mass in overweight or obese adults. J Appl Physiol (1985) 2012;113:1831-7. doi: 10.1152/japplphysiol.01370.2011
